# Myofibroblasts Electrotonically Coupled to Cardiomyocytes Alter Conduction: Insights at the Cellular Level from a Detailed *In silico* Tissue Structure Model

**DOI:** 10.3389/fphys.2016.00496

**Published:** 2016-10-27

**Authors:** Florian Jousset, Ange Maguy, Stephan Rohr, Jan P. Kucera

**Affiliations:** Department of Physiology, University of BernBern, Switzerland

**Keywords:** action potential propagation, cardiac tissue, myofibroblasts, slow conduction, computer simulation, cardiac fibrosis, electrotonic interactions

## Abstract

Fibrotic myocardial remodeling is typically accompanied by the appearance of myofibroblasts (MFBs). *In vitro*, MFBs were shown to slow conduction and precipitate ectopic activity following gap junctional coupling to cardiomyocytes (CMCs). To gain further mechanistic insights into this arrhythmogenic MFB-CMC crosstalk, we performed numerical simulations in cell-based high-resolution two-dimensional tissue models that replicated experimental conditions. Cell dimensions were determined using confocal microscopy of single and co-cultured neonatal rat ventricular CMCs and MFBs. Conduction was investigated as a function of MFB density in three distinct cellular tissue architectures: CMC strands with endogenous MFBs, CMC strands with coating MFBs of two different sizes, and CMC strands with MFB inserts. Simulations were performed to identify individual contributions of heterocellular gap junctional coupling and of the specific electrical phenotype of MFBs. With increasing MFB density, both endogenous and coating MFBs slowed conduction. At MFB densities of 5–30%, conduction slowing was most pronounced in strands with endogenous MFBs due to the MFB-dependent increase in axial resistance. At MFB densities >40%, very slow conduction and spontaneous activity was primarily due to MFB-induced CMC depolarization. Coating MFBs caused non-uniformities of resting membrane potential, which were more prominent with large than with small MFBs. In simulations of MFB inserts connecting two CMC strands, conduction delays increased with increasing insert lengths and block appeared for inserts >1.2 mm. Thus, electrophysiological properties of engineered CMC-MFB co-cultures depend on MFB density, MFB size and their specific positioning in respect to CMCs. These factors may influence conduction characteristics in the heterocellular myocardium.

## Introduction

Cardiac tachyarrhythmias based on re-entrant excitation are serious heart rhythm disorders associated with a variety of heart diseases. In re-entrant arrhythmias, the electrical excitation wave (action potential, AP) continuously re-excites myocardial regions that have recovered from refractoriness. This self-perpetuating process is independent of the natural pacemaker of the heart, the sinoatrial node. Mechanistically, the induction of a re-entrant activity depends on conduction block and slow conduction (Kléber and Rudy, 2004). Conduction block, in particular unidirectional block, prevents excitation of defined regions within the myocardium during the priming activation phase. Conduction blocks contribute to ectopic activity and were shown to increase the susceptibility to atrial fibrillation (Haïssaguerre et al., 1998; Chen et al., 1999). Additionally, slow conduction around the site of block will, if sufficiently delayed for the tissue proximal to the block to recover from refractoriness, cause re-excitation thereby initiating re-entry.

It is well established that conduction velocity and occurrence of conduction blocks are determined by (i) the level of gap junctional coupling between adjacent cardiac cells and (ii) the membrane currents shaping the AP with voltage gated inward currents being particularly important (Kléber and Rudy, 2004). The disruption of the normally uniform cardiac tissue architecture during cardiac fibrotic remodeling is well established to slow conduction and promote conduction blocks due to the presence of electrically insulating sheets of collagen (Camelliti et al., 2005).

Myofibroblasts (MFBs, “activated fibroblasts”) are held responsible for the excessive secretion of extracellular matrix proteins during fibrotic remodeling. They were shown *in vitro* to slow conduction and precipitate ectopic activity following establishment of heterocellular electrotonic coupling to cardiomyocytes (CMCs) (Miragoli et al., 2006, 2007). While the origin of MFBs in the working myocardium is still under debate, it is generally assumed that they emerge from resident cardiac fibroblasts undergoing a phenotype switch to MFBs secondary to insults to the heart like pressure/volume overload and infarction. MFBs are identified by their expression of α-smooth muscle actin decorated stress fibers. They are typically larger than fibroblasts and produce excess amounts of extracellular matrix proteins like collagen, thereby contributing to the normal process of tissue repair but also to pathological fibrotic remodeling that leads to conduction disturbances by disrupting the myocardial tissue architecture (Camelliti et al., 2005; Rohr, 2009). When fibroblasts/MFBs connect electrically to CMCs, they can then form single-sided connections to one functionally connected group of CMCs or double-sided connections to separate groups of CMCs (Kohl and Camelliti, 2012).

In previous work (Gaudesius et al., 2003), we showed that MFBs inserted into gaps in patterned CMC strands allow the passage of sufficient depolarizing current to support resumption of conduction distal to the insert. Conduction blocks occurred only when insert lengths exceeded several hundred micrometers. In another study (Miragoli et al., 2006), we showed that MFBs (either interspersed within CMC strands or seeded on top of CMC strands at predefined densities) exert a depolarizing effect on electrotonically connected CMCs, thereby causing conduction slowing. Furthermore, we found in monolayer cultures of CMCs that MFBs induce regular ectopic activity in a cell-density dependent manner (Miragoli et al., 2007).

Previous computer simulations of cardiac tissue have corroborated the pro-arrhythmic effects of fibroblasts (Jacquemet and Henriquez, 2008; Sachse et al., 2008; Maleckar et al., 2009). Using a microstructure model of cardiac tissue, Jacquemet and Henriquez showed that fibroblasts with one-sided connections exert non-linear effects on resting membrane potential, conduction velocity and action potential duration that depend in a complex manner on fibroblast density, fibroblast capacitance and CMC-fibroblast coupling (Jacquemet and Henriquez, 2008). Using a computational framework permitting to combine rectangular CMCs and square-shaped fibroblasts in a brick-like manner, Xie et al. additionally investigated the influence of fibroblasts with double-sided connections (Xie et al., 2009a). By exploring a broad range of fibroblast densities and CMC-fibroblast coupling values, they observed that with increasing coupling, fibroblasts with double-sided connections can first accelerate conduction by depolarizing the CMCs (supernormal conduction), then slow conduction due to their increasing electrotonic loading effect, before finally accelerating conduction again by acting as conductive bridges between CMCs. Nayak et al. investigated the effects of one-sided and two-sided CMC-fibroblast coupling on spiral wave dynamics without describing the detailed pattern of cellular arrangements in their model (Nayak et al., 2013). Kim et al. developed a high resolution model of engineered CMC monolayers permitting to simulate realistic cell shapes and showed that conduction characteristics are influenced by the structure of the tissue microarchitecture (Kim et al., 2010). However, the effects of CMC coupling to fibroblasts were not explored in their study.

To our knowledge, there exists no study that uses a high resolution cardiac tissue model implementing realistic cell shapes for both CMCs and MFBs that permits a direct comparison with former experimental findings. Under the tenet that CMCs and MFBs are electrotonically coupled in the myocardium, the mechanistic characterization of previous experimental findings with a high-resolution tissue model is highly relevant insofar as that the detailed cellular architecture of cardiac tissue consisting of CMCs and MFBs and the resulting microconduction patterns are likely to play a major role in determining the specific characteristics of conduction slowing, conduction block and precipitation of ectopic activity which are fundamental for the generation and perpetuation of re-entrant arrhythmias.

Accordingly, it was the aim of the present study to develop a mathematical model of cardiac impulse conduction on the basis of a detailed description of the heterogeneous cellular architecture of tissues consisting of CMCs and MFBs. The model was developed to match as closely as possible the experimental preparations that we used previously. The model was validated with *in vitro* measurements of preparations with known cellular architecture. We investigated the following three distinct tissue architectures: (i) CMC strands containing endogenous MFBs, (ii) CMC strands coated with MFBs, and (iii) CMC strands containing an intercalated segment consisting of MFBs. For CMC strands coated with MFBs, we explored the effects of two different sizes of MFBs. Because the ion current repertoires of different cell types and heterocellular gap junctional coupling can be individually controlled in the model, simulations allowed untangling the relative contributions of each of these factors to the observed electrical phenotypes of these preparations.

## Materials and methods

### *In-vitro* fibrosis model

#### Cell culture

Primary cultures of neonatal rat ventricular CMCs and MFBs were obtained using methods described in detail before (Rohr et al., 2003). Protocols were in agreement with relevant institutional guidelines for animal experimentation and were approved by the Veterinary Office of the Canton of Bern, Switzerland. Briefly, the apical two thirds of 1-day old rat ventricles were cut in small pieces and dissociated in Hanks' balanced salt solution (HBSS without Ca^2+^ and Mg^2+^, Bioconcept) containing trypsin (0.1%, Sigma) and pancreatin (120 μg/ml, Sigma). Tissue digestion was complete following 4–5 dissociation cycles lasting 15 min each. After each cycle, tissue pieces were left to sediment and the supernatant, containing the dissociated cells, was removed and stored on ice. The dissociated cells were centrifuged and re-suspended in culture medium consisting of M199 with Hanks' salts (Sigma), penicillin (20,000 U/L; Sigma), vitamin B12 (1.5 μmol/L, Sigma) and 10% neonatal calf serum (NCS, Biochrom, Bioswisstec). Fast adhering fibroblasts were separated from slowly adhering CMCs by preplating the cell suspension for 2 h 15 min in 75 cm^2^ cell culture flasks. The supernatant containing mostly cardiomyocytes was collected, passed through a cell strainer and supplemented with vitamin C (18 μmol/L, Sigma), epinephrine (10 μmol/L, Sigma) and bromodeoxyuridine (100 μmol/L, Sigma). After counting the cells, appropriate dilution of the suspension was made. Cells were plated onto different types of culture substrates (cf. below). Medium exchanges were performed 24 h post-plating with supplemented M199 containing a reduced amount of NCS (5%) and every 48 h thereafter. Fibroblasts obtained from the pre-plating procedure were thoroughly washed to remove non-attached or weakly attaching cells and were kept in supplemented medium without bromodeoxyuridine. Cultured fibroblasts expressed α-smooth muscle actin (α-SMA) within 24–48 h indicating a rapid phenotype switch to MFBs (Rohr, 2011). MFBs were kept in culture for 8 days prior to being used in the experiments. Cell cultures were maintained at 35°C in a humidified atmosphere containing 1.2% CO_2_.

##### Single cell culture

For confocal imaging of single cells, CMCs or MFBs were seeded on collagen (Type I or Type IV, Sigma) coated glass coverslips at 40–80 cells/mm^2^ which resulted in low density cultures. Cell culture media and medium exchange protocols were identical to those described above.

##### Patterned growth cell cultures

Strands of CMCs measuring 0.6 × 5 mm were obtained by seeding CMCs at a density of 1500 cells/mm^2^ onto photolithographically generated strips of collagen type IV (Sigma) as described in detail before (Rohr et al., 2003). For models of cardiac fibrosis, 24 h old CMC strands were coated with MFBs at a density of 500 cells/mm^2^.

#### Fluorescent staining of cellular preparations

For single cell live imaging, MFBs and CMCs were stained with Vybrant™ DiI (Molecular Probes). Cells were washed twice with HBSS before being stained with Vybrant™ DiI (5 μl of stock solution diluted in 1 ml HBSS) for 20 min in the incubator. Thereafter, cells were washed three times with HBSS to prevent non-specific binding of the dye and reduce background fluorescence.

Imaging of CMC strands with endogenous or coating MFBs was performed on immuno-stained preparations. Pattern-growth cultures were washed twice with HBSS and fixed with 2% paraformaldehyde for 10 min. After a second washing step, strands were incubated for 20 min with a blocking buffer (PBS containing 20% goat serum) followed by a 2 h exposition to anti α-SMA antibodies (mouse monoclonal, Thermo Fisher) dissolved in PBS containing 1% goat serum and 0.15% Triton X-100. After washing, preparations were incubated for 20 min with a goat anti-mouse AlexaFluor 488 secondary antibody (Molecular Probes). Preparations were further washed and finally mounted. All steps were carried out at room temperature.

#### Confocal imaging and post-processing

**Figure 1:**
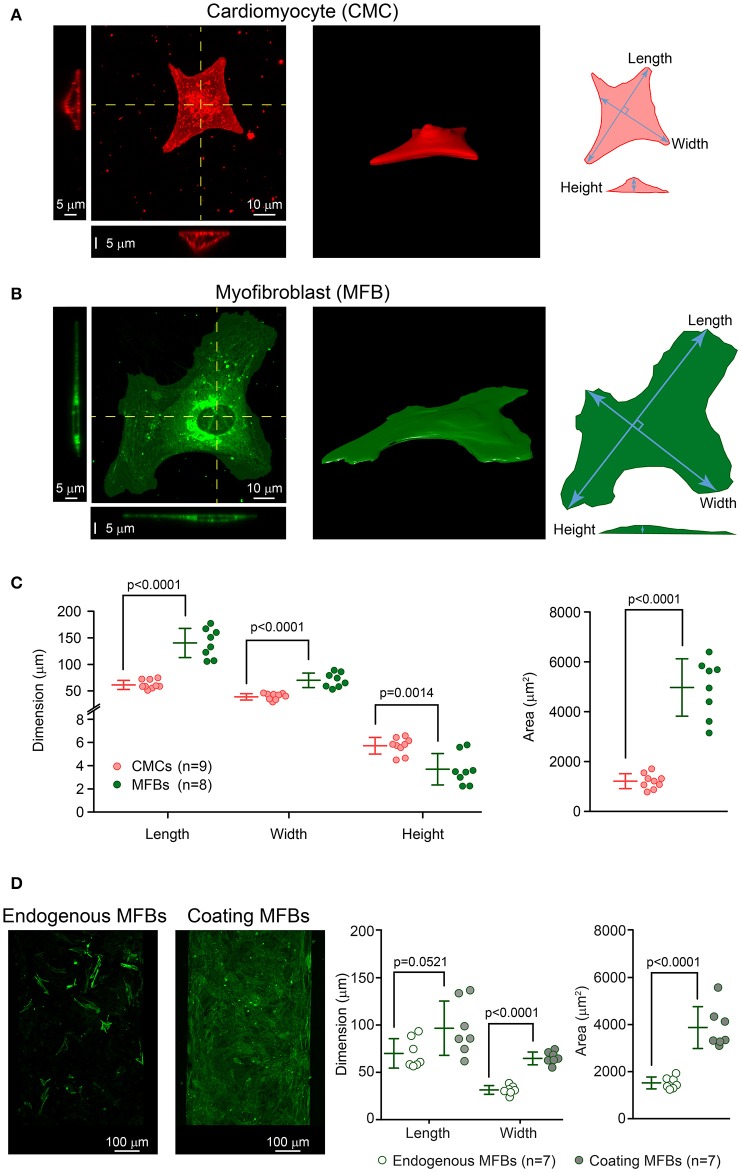
**Dimensions of cardiomyocytes (CMCs) and myofibroblasts (MFBs)**. **(A)** Example of a single cultured CMC: top view with two projections along the yellow lines (left), 3D reconstruction (middle) and schematic of the measurements (right). **(B)** Same as A for a single cultured MFB. **(C)** Left panel: length, width and height of single CMCs (pink, *n* = 9) and MFBs (green, *n* = 8) with mean ± SD. Right panels: dito for cell surface area. **(D)** Images: representative examples of CMC strands with endogenous and coating MFBs. Right panels: Dimensions and surface areas of endogenous MFBs (*n* = 7) and coating MFBs (*n* = 7) with mean ± SD.

Image acquisition was performed on an inverted laser scanning microscope (LSM 880 using the ZEN 2.1 software, Zeiss). Single cell imaging was performed with Airyscan in high resolution mode using a Plan Apochromat 40x objective (NA1.3, Oil DIC, Zeiss). Live-stained CMCs and MFBs were imaged with a 2.4 or 1.8 zoom (pixel size: 165 and 220 nm, respectively). Acquisition was made in line scan mode with an averaging factor of 2. Z-stacks of entire cells were acquired at a resolution of 150 nm. TIFF image series were exported to ImageJ software (National Institutes of Health) for post-processing. Maximum length, width and area of cells were determined from maximum intensity full projections as illustrated in Figures 1A,B. The height of the cells was obtained from orthogonal sections. IMOD (Boulder laboratory) was used for segmentation and 3D reconstitution of the cells. Rendering was performed with Rhinoceros 5.0 (McNeel). Fixated and immunostained cell strands were imaged using a Plan Apochromat 10x objective (NA 0.45, Zeiss) at a pixel size of 815 nm. Acquisition was made in line scan mode with an averaging factor of 2. Z-stacks covering the entire thickness of the preparations were acquired with a step size of 410 nm. TIFF image series were exported to ImageJ and cellular morphometry of endogenous or coating MFBs was performed on maximum intensity full projections (Figure 1D).

### Tissue model

#### CMC strands with endogenous and coating MFBs

Following our previous approach (Prudat and Kucera, 2014), simulations were based on randomly seeded cellular networks mimicking the irregular layout of the cells in two-dimensional layers of CMCs or CMCs and MFBs. Briefly, the domains to be simulated were partitioned into polygons describing individual cells. The polygons were then discretized using a regular grid. Our model produces tissue microstructures that are similar to those generated by the procedures developed by Jacquemet and Henriquez (2008) and by Kim et al. (2010), although the underlying algorithms are different.

**Figure 2:**
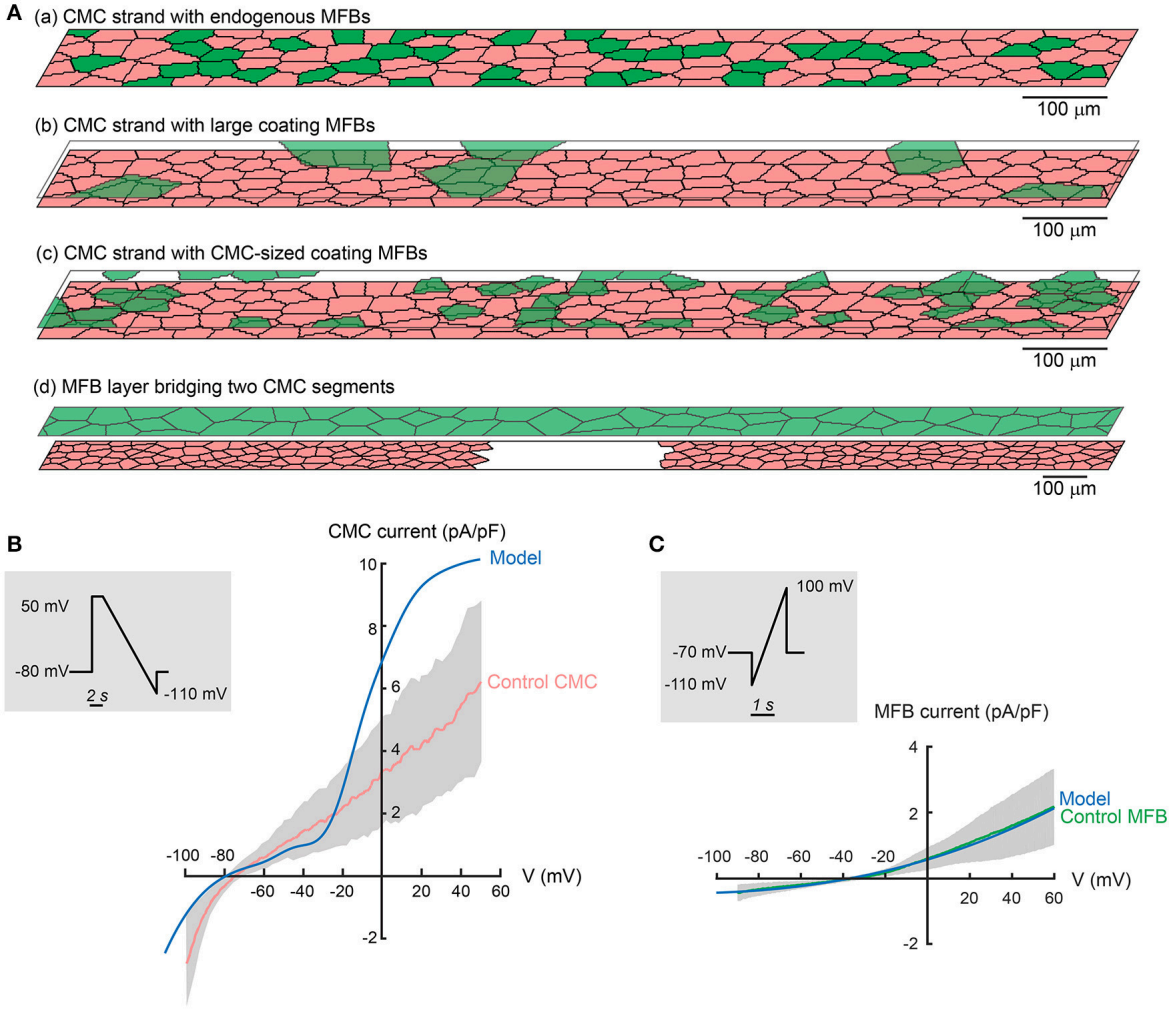
**Tissue and ionic models. (A)** Schematic representation of tissue models: **(a)** strand of CMCs (pink) with endogenous MFBs (green); **(b)** CMC strand with large coating MFBs; **(c)** CMC strand with CMC-sized coating MFBs; **(d)** CMC strand disrupted by a gap and fully covered by a layer of MFBs bridging the gap (MFB insert). **(B)** Comparison of experimentally measured I–V relationship of CMCs (pink/gray corresponds to mean ± SD, *n* = 12) to the modified LR1 CMC model (blue) subjected to the same ramp protocol (insert). **(C)** I–V relationship measured in MFBs (green/gray corresponds to mean ± SD, *n* = 11) subjected to the protocol shown in the inset. The fitted model function (Equation 6) is shown in blue. Experimental data from Grand et al. (2014).

MFBs were either embedded within (endogenous MFBs) or seeded on top (coating MFBs) of the CMC layer, as illustrated in Figures 2Aa–c. To ensure consistency with our experimental data, the cellular model was adapted as to reflect typical cell dimensions of MFBs and CMCs in our cultured preparations.

In accordance with the dimensions of CMC patterned growth strands used in previous experimental work, cellular networks consisted of two-dimensional domains measuring 3000 × 80 μm (Gaudesius et al., 2003; Miragoli et al., 2006). Based on own past work (Rohr et al., 1991) and morphometric findings (Figures 1A–C), the average CMC length and width were set to 60 and 20 μm, respectively, resulting in a length/width ratio of 3. Confocal microscopy performed on similarly sized single cultured cells showed a median maximal thickness of 5.7 μm (CMCs) and 3.7 μm (MFBs). In our simulated strands, the average thickness of both CMCs and MFBs was then set to 3 μm. The CMC networks with endogenous MFBs were discretized using a square lattice with a spatial step of Δx = Δy = 2.5 μm. The CMC networks with coating MFBs were discretized using a tetragonal lattice with a spatial step of Δx = Δy = 2.5 μm and Δz = 3 μm, with Δz corresponding to the average cell thickness.

The morphometric data presented in Figure 1 show that endogenous MFBs are smaller than coating MFBs and close to the individual size of CMCs. Therefore, the dimensions of endogenous MFBs were set to be similar to those of CMCs. As aforementioned, coating MFBs are typically 1.3x longer and 2x wider, with an area reaching 2.5x that of endogenous MFB (Figure 1D). This observation was supported by the analysis of MFB dimensions in datasets from previous studies (Miragoli et al., 2006; Grand et al., 2014). Our model was implemented accordingly to consider the extreme situation with MFBs having a length and a width of 120 and 40 μm, i.e., twice that of CMCs. In simulations aiming to investigate the effects of MFB size on conduction, the dimensions of coating MFBs were alternatively set to that of endogenous MFBs (CMC-sized MFBs, Figure 2Ac). Cell dimensions used in the simulations are recapitulated in Table 1.

**Table 1:** **Cellular dimensions and passive resistive and capacitive parameters of the modeled CMCs and MFBs**.

**Parameter**	**Definition**	**Value**			**References**
		**CMC**	**CMC-sized MFB**	**Large MFB**	
**MEASURED PARAMETER**					
*L*	Cell length	0.0060 *cm*	0.0060 *cm*	0.0120 *cm*	Rohr et al., 1991
*W*	Cell width	0.0020 *cm*	0.0020 *cm*	0.0040 *cm*	Rohr et al., 1991
*T*	Cell thickness	0.0003 *cm*	0.0003 *cm*	0.0003 *cm*	
ρ_*cyto*_	Cytoplasmic resistivity	124 Ω *cm*	124 Ω *cm*	124 Ω *cm*	Prudat and Kucera, 2014
*C_m_*	Specific membrane capacitance	1 μ*F*/*cm*^2^	1 μ*F*/*cm*^2^	1 μ*F*/*cm*^2^	
		CMC-CMC	CMC-MFB	MFB-MFB	
γ	Intercellular conductivity	77.4 *nS*/μ*m*	15.5 *nS*/μ*m*	15.5 *nS*/μ*m*	Fitted
**DERIVED PARAMETER**					
ρ	Intercellular resistivity (1/γ)	1351 Ω *cm*	6755 Ω *cm*	6755 Ω *cm*	
ρ_*cyto, z*_	Cytoplasmic resistivity in the *z* direction	*T*·ρ_*cyto*_	37.2·10^−3^Ω*cm*^2^	37.2·10^−3^Ω*cm*^2^	
*r_cyto, z_*	Cytoplasmic resistance per discrete element in the *z* direction	$\frac{T\cdot \rho_{cyto}}{\Delta x\Delta y}$	0.595 *MΩ*	0.595 *MΩ*	
ρ_*gap, z*_	Gap junctional resistivity in the *z* direction	$\frac{\rho }{L}\pi \frac{LW}{4}$	21.2 *GΩ cm*^2^	106.1 *GΩ cm*^2^	
*r_gap, z_*	Gap junctional resistance per discrete element in the *z*-direction	$\frac{\rho }{L}\;\pi \frac{LW}{4\Delta x\Delta y}$	0.339 *GΩ*	1.698 *GΩ*	
		CMC-CMC	CMC-MFB	MFB-MFB	
*g_z_*	Conductance per discrete element in the z-direction $\left(\frac{1}{r_{cyto,z}+r_{gap,z}}\right)$	2.94 *nS*	0.59 *nS*	0.59 *nS*	

Simulations were run at different MFB to CMC ratios. To compare results between simulations with endogenous MFBs and coating MFBs, we defined the parameter “MFB density” (%MFB) which represents the relative contribution of MFBs to the total tissue capacitance. MFB density was computed as the ratio of the area occupied by MFBs to the total tissue area. For the situation of coating MFBs, this ratio was computed as *c*/(1+*c*), where *c* is the MFB coverage ratio. For each MFB density, conduction was simulated in 5 independently generated tissues.

#### CMC strands with MFB inserts

CMC strands with MFB inserts were designed as to reproduce tissue models used previously to investigate conduction across segments of unexcitable MFBs (Gaudesius et al., 2003). CMC strands measuring 6000 × 80 μm were interrupted in the center over a predefined distance (240–2880 μm). The strand segments and the gap were subsequently coated with MFBs (full coverage) resulting in a link of the two CMC segments by MFBs (Figure 2Ad). For each MFB insert length, conduction was simulated in 5 independently generated tissues.

#### Passive electrical parameters

##### Cytoplasmic resistivity

Cytoplasmic resistivity was set to 124 Ωcm (Prudat and Kucera, 2014). This value is in the range of resistivities of physiological electrolyte solutions and matches values used in past computational studies (100–180 Ωcm; Kléber and Riegger, 1987; Shaw and Rudy, 1997; Hubbard and Henriquez, 2012). Cytoplasmic resistivity was assumed to be isotropic and identical for CMCs and MFBs.

##### Gap junctional conductivity

As done previously in murine ventricular cell culture models (Prudat and Kucera, 2014), gap junctional conductance between CMCs was adjusted conjointly with the voltage gated sodium current density to replicate the conduction velocity (CV) and the rate of rise of the action potential (dV/dt_max_) observed in experiments (Rohr et al., 1998). Intercellular gap junctional conductance was proportional to the contact length between neighboring CMCs and was thus represented by a proportionality constant, γ, representing conductivity (conductance per contact length, see Table 1). The total gap junctional conductance between two adjacent cells (G) was computed as *G* = γ·L_c_, where L_c_ is the length of contact computed from the polygons describing the cells before discretization. After discretization, oblique contacts then assume a staircase shape with an increased length. To compensate the increased length caused by discretization, the discrete conductances (g) across individual staircase segments were computed as *g* = G/N, with N being the number of staircase segments. The total intercellular gap junctional coupling conductance was therefore redistributed uniformly along the contact.

The gap junctional conductivity between CMCs and MFBs (CMC-MFB) was set to be 5 times smaller than between CMCs (Salvarani et al., 2012). We further assumed that the conductivity between MFBs is the same as that between a CMC and a MFB. This assumption relies on the fact that each cell has to contribute one hemichannel for the formation of one functional connexon, and that intercellular junctional conductivity is thus determined by the cell expressing the lowest amount of connexins.

In monolayer cultures immunostained for connexin 43, it was observed that the intensity of the staining does not depend on the orientation (transverse vs. longitudinal) of the intercellular contact relative to the cell axis (Gaudesius et al., 2003; Miragoli et al., 2006). Therefore, intercellular junctional conductivity was assumed to be isotropic in the model (γ did not depend on the orientation of the intercellular contacts).

For intercellular conductivities between the layer of CMCs and coating MFBs (in the z direction), we assumed that, on average, the number of junctional channels between a CMC and a coating MFB is the same as between a CMC and a longitudinally connected endogenous MFB. This parameter was therefore calculated based on cell length and the area of a typical elliptical cell shape (Table 1).

##### Cell capacitance

The specific membrane capacitance of CMCs and MFBs was set to 1 μF/cm^2^ (Table 1). The cell capacitance results from the product of the specific membrane capacitance and the geometrical area (surfaces above and below the cell as well as the lateral surface), leading to a mean CMC capacitance of 22.86 pF and a mean capacitance per monolayer unit area of 2.43 μF/cm^2^. Endogenous MFBs have the same capacitance as CMCs and large coating MFBs have a mean capacitance of 83.42 pF.

#### Ionic currents of CMCs

CMC ion currents were represented using the Luo-Rudy I (LR1) model (Luo and Rudy, 1991) which was adjusted to approximate our experimental observations in single cultured CMCs subjected to voltage clamp experiments shown in Figure 2B. Compared to these data, the inward rectifier K^+^ current (I_*K*1_) of the original LR1 model (not shown) overestimated several fold the measured I_*K*1_ near the resting membrane potential. Because I_*K*1_ is particularly important in determining the resting membrane potential and input resistance of CMCs, the formulation of I_*K*1_ of the LR1 model was therefore replaced by the I_*K*1_ formulation of Korhonen et al. (2009) provided in Equation (1), which is based on data recorded in neonatal rat ventricular myocytes by Wahler (1992).

(1) $$\begin{array}{l} I_{K1}\;=\;0.0515\cdot \frac{{\left[K^{+}\right]}_{o}}{{\left[K^{+}\right]}_{o}+0.210}\cdot \frac{V-E_{K1}-6.1373}{0.1653+e^{0.0319\left(V-E_{K}-6.1373\right)}} \end{array}$$

In presence of this reduced *I*_*K*__1_, the background current (I_b_) caused a substantial and non-physiological shift of the resting membrane potential to more positive values and was therefore set to 0. In the range from −80 to −60 mV, which corresponds to the RMP of CMCs coupled to MFBs, the current-voltage curve of the CMC model with the *I*_*K*__1_ formulation of Korhonen et al. lies within the mean ± 1 standard deviation as found in experiments. The *I*_*K*__1_ formulation of Korhonen et al. thus reproduces our measurements.

For the Na^+^ current, I_Na_, we used the LR1 formulation with maximal Na^+^ current conductance (g_Na, max_) being adjusted such as to replicate conduction velocity (CV) and the maximal rate of rise of the AP upstroke as described above and in Table 1. In CMC networks, setting the intercellular conductivity to 77.4 nS/μm and g_Na, max_ to 8.1 resulted in a CV of 41.4 cm/s and a dV/dt_max_ of 106.8 V/s which closely reproduced findings obtained during optical recordings in patterned growth CMC strands (Rohr et al., 1998).

It is known that the slow inward Ca^2+^ current, I_CaL_, plays an important role in the presence of very slow conduction or local conduction delays exceeding the typical timescale of I_Na_ (Rohr and Kucera, 1997; Shaw and Rudy, 1997). Therefore, as in previous work (Prudat and Kucera, 2014), we increased the maximal conductance of I_CaL_ from 0.09 to 0.18 mS/μF and accelerated its gating kinetics [rate constants of the activation gate *d* multiplied by 30, rate constants of the inactivation gate *f* multiplied by 2, Equations (2–5)]. The adjusted I_CaL_ exhibited a time to peak of ~1 ms, comparable to previous experimental and modeling studies (Shaw and Rudy, 1997; Linz and Meyer, 2000; Wang and Sobie, 2008; Prudat and Kucera, 2014; Jousset and Rohr, 2015). We note that the role of I_CaL_ in sustaining propagation during a reduction of coupling becomes important only when intercellular coupling is decreased by >90% (Shaw and Rudy, 1997). In the present work, intercellular coupling between CMCs and MFBs was 20% of CMC-CMC coupling. At 20% of CMC-CMC coupling level, the safety factor for propagation is not manifestly different in presence vs. absence of I_CaL_ (Shaw and Rudy, 1997).

We observed that I_CaL_ causes spontaneous activity in the CMC membrane model when using the I_K1_ formulation of Korhonen et al. This spontaneous activity is caused by a large steady state “window” current which produces a downward inflection (minimum near −35 mV) of the steady state I–V curve of the CMC ionic model. This window inward Ca^2+^ current in turn causes the steady state I–V curve not to cross the abscissa (*I* = 0) in the expected resting membrane potential range thereby leading to an unstable potential and spontaneous activity. In contrast to this behavior, dense monolayer cultures of CMCs are quiescent and become spontaneously active only when coupled to a sufficient number of MFBs (Miragoli et al., 2007). To accommodate for this difference, the window current was decreased by shifting the activation and deactivation rate constants of the activation gate *d* by 6 mV toward more positive potentials which is in accordance to experimental data (Xiao et al., 1997; Pignier and Potreau, 2000). The modified rate constants of the gating variables *d* and *f* are given by Equations (2–5):

(2) $$\begin{array}{r} \alpha_{d}=30\frac{0.095e^{-0.01\left(\left(V-6\right)-5\right)}}{1+e^{-0.072\left(\left(V-6\right)-5\right)}} \end{array}$$

(3) $$\begin{array}{r} \beta_{d}=30\frac{0.07e^{-0.017\left(\left(V-6\right)+44\right)}}{1+e^{0.05\left(\left(V-6\right)+44\right)}} \end{array}$$

(4) $$\begin{array}{r} \alpha_{f}=2\frac{0.012e^{-0.008\left(V+28\right)}}{1+e^{0.15\left(V+28\right)}} \end{array}$$

(5) $$\begin{array}{r} \beta_{f}=2\frac{0.0065e^{-0.02\left(V+30\right)}}{1+e^{-0.2\left(V+30\right)}} \end{array}$$

This modification caused the steady state I–V curve to cross the abscissa near −79 mV with a positive slope, thereby leading to a stable resting membrane potential (RMP) at this value. Finally, to account for short APs typical for small rodents, the maximal conductance of the voltage gated K^+^ current (I_*K*_) was increased from 0.282 to 0.5 mS/μF. The plateau K^+^ current was not modified. The steady state I-V curve of the modified CMC model is shown in Figure 2B.

#### Ionic currents of MFBs

We formulated our own MFB model based on data from voltage clamp experiments with cultured cardiac MFBs (Figure 2C; Grand et al., 2014). As illustrated in Figure 2C, the mean I–V relationship of MFBs was fitted with the following function of voltage (Equation 6):

(6) $$\begin{array}{l} I_{MFB}\;=\;-12.5-0.033\cdot V+\exp\left(\frac{V+642.5}{250}\right) \end{array}$$

The RMP of the isolated MFB model was −36.1 mV.

#### Numerical methods

Because culture experiments are performed in an extended extracellular medium, the resistance of the extracellular space was considered negligible and a monodomain formulation was adopted (Prudat and Kucera, 2014).

The generation of the networks was implemented in MATLAB (version 2015b, The MathWorks, Natick, MA) with the desired configuration of the network (i.e., size, MFB density) and the different parameters of the model (Table 1) serving as input. The program returned the layout of the network and the computed resistances between adjacent nodes in the discretized lattice as described in detail before (Prudat and Kucera, 2014).

To simulate electrical activity, a fixed time step Δ*t* of 0.005 ms was used. The gating variables of the adjusted LR1 model were integrated using the method of Rush and Larsen (1978) and the intracellular concentration of Ca^2+^ was integrated using the forward Euler method. These computations were implemented in the C language and executed in the MATLAB environment as dynamically linked subroutines (MEX files). The determination of the opening and closing rate constants of the different ion current gates was optimized for speed by computing tabulated look-up tables at the beginning of each simulation.

The diffusion problem (the change of membrane potential caused by current flux to and from adjacent nodes across a heterogeneous network of resistances) was solved using the method of Crank and Nicolson (1947). This method was implemented in matrix form as a linear system of *N* equations with *N* unknowns, *N* being the number of nodes in the simulated domain (144,000 or more in our study), and formulated as:

(7) $$\begin{array}{l} \left(\text{I}-\frac{1}{2}\Delta \text{t}\text{A}\right)\text{v}\left(t+\Delta t\right)=\left(\text{I}+\frac{1}{2}\Delta \text{t}\text{A}\right)\text{v}\left(t\right) \end{array}$$

where ***I*** is the identity matrix, *v*(*t*) is the known vector of potentials at time *t*, *v*(*t* + Δ*t*) is the unknown vector of potentials to be solved for time *t* + Δ*t* and ***A*** is a *N* × *N* sparse symmetric matrix. The entries of ***A*** are given by the passive resistive and capacitive properties of the network, which do not change with time. The system was solved by performing the Cholesky decomposition (Press et al., 1997) of the left hand operator (***I***–Δt***A***/2) as

(8) $$\begin{array}{l} \text{I}-\frac{1}{2}\Delta \text{t}\text{A}\;=\;\text{L}\cdot {\text{L}}^{T} \end{array}$$

where ***L*** is a lower triangular matrix and ***L**^T^* is the transpose of *L*. The solution to Equation (7) was thus decomposed in two steps consisting of solving two linear systems given by a triangular matrix (***L*** or ***L**^T^*).

To compute ***L*** and ***L**^T^*, we took advantage of the advanced Cholesky decomposition algorithms optimized for sparse matrices as incorporated in MATLAB. Since a fixed Δt was used, ***L*** and ***L**^T^* needed to be computed only once at the beginning of each simulation. These matrices were passed to the core C program, in which the algorithm to solve triangular systems was implemented.

#### Simulation protocol

The strands with endogenous or coating MFBs were first simulated without any electrical stimulation for at least 2 s to examine whether any spontaneous activity was present and to allow for steady state equilibration. The network was then stimulated by injecting a current pulse into the cells at one extremity of the strand. The following parameters were registered for every action potential at every node: (1) RMP, defined as membrane potential at the time of stimulation, (2) maximal rate of rise of the AP upstroke (dV/dt_max_), and (3) activation time that was defined as the time at which membrane potential reached −35 mV during the action potential upstroke. Conduction velocity (CV) was computed by linear regression of the earliest activation time at each *x*-coordinate between 25 and 75% of strand length to exclude stimulation artifacts and sealed-end effects at the extremities of the strands.

For the MFB inserts simulations, the strands were stimulated at 2 Hz and electrophysiological parameters were extracted from the AP elicited by the 6th stimulation.

#### Statistical analysis

Normality of distribution was assessed using the Shapiro-Wilk test. Normally distributed datasets are expressed as mean ± standard deviation (SD) and were compared with Student's non-paired *t*-test.

## Results

### Slow conduction in CMC strands with endogenous MFBs

**Figure 3:**
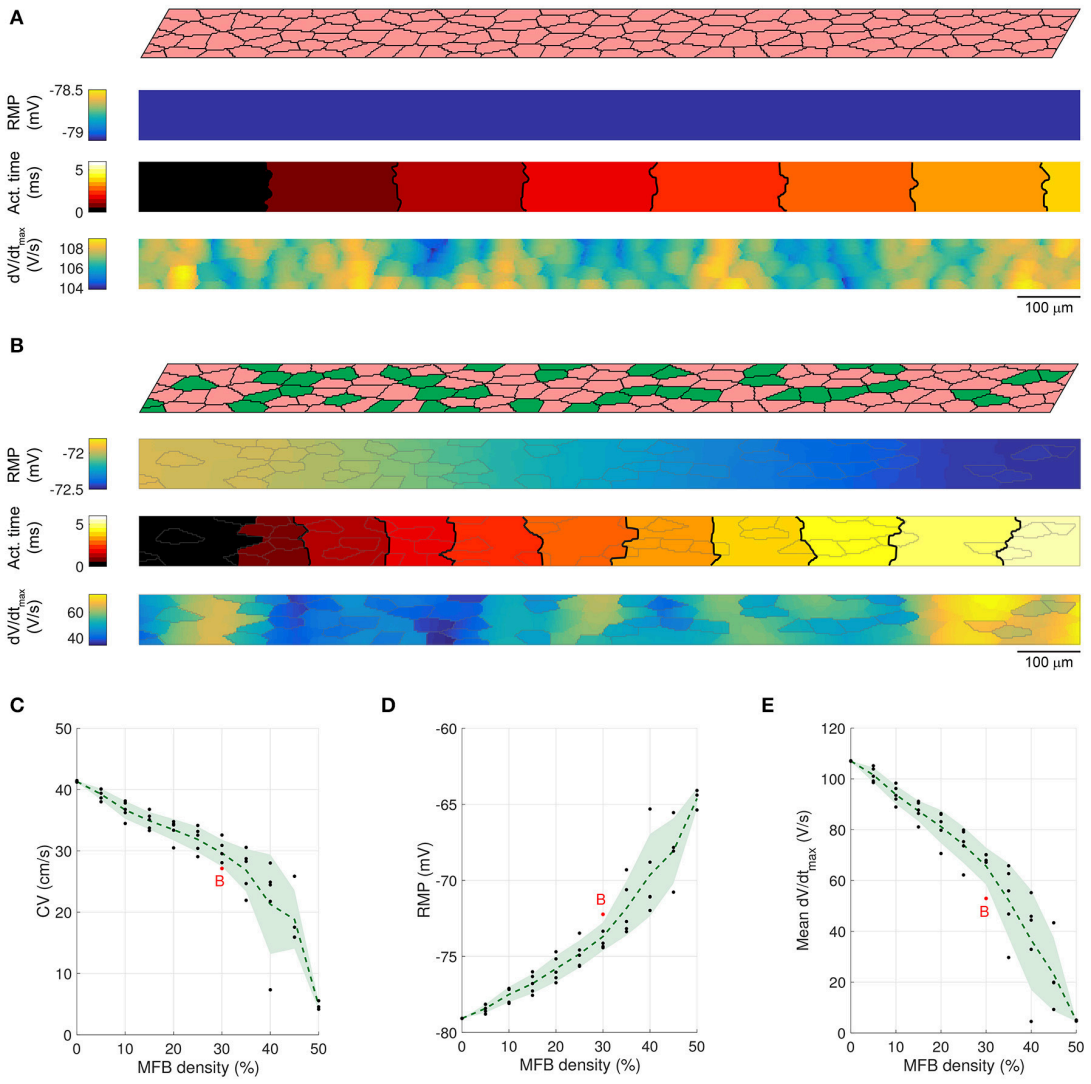
**Conduction in control CMC strands and CMC strands with endogenous MFBs**. **(A)** Structure of a computer-generated control CMC strand (100% of CMCs, pink) and corresponding maps of RMP, activation time (isochrone interval: 0.5 ms) and dV/dt_max_. **(B)** Same as A for a CMC strand with endogenous MFBs (MFBs density: 30%, green). In the maps, MFB cell borders are outlined in gray. **(C–E)** Effects of increasing MFB density on conduction velocity, mean RMP and mean dV/dt_max_ (black: individual results; green: mean ± SD, *n* = 5; red data point corresponds to the simulated tissue shown in **B**).

In previous *in vitro* experiments, we showed that endogenous MFBs slow conduction in CMC strands in an MFB-density dependent manner (Miragoli et al., 2006). This situation was simulated by generating 3000 × 80 μm CMC networks containing endogenous MFBs at densities ranging from 0 to 50% in steps of 5%. In accordance with the *in vitro* experiments, CMCs and endogenous MFBs had the same size. Figures 3A,B illustrate AP propagation in the central part (25–75%) of a control strand (CMCs only) and in a strand containing 30% endogenous MFBs. In the control strand (Figure 3A), RMP was uniform and propagation was quasi continuous as demonstrated by the regular spacing of activation isochrones which were oriented perpendicular to the strand axis. Slight distortions of isochrones and small fluctuations of dV/dt_max_ in the range of a few percent were the consequence of the underlying discrete cellular architecture with variable local differences in intercellular resistance. In the strand containing 30% MFBs (Figure 3B), RMP was depolarized by ~6 mV compared to the control CMC strand. Gap junctional coupling strongly attenuated the differences in RMP between CMCs and MFBs. In the example shown, the small RMP gradient present was due to the incidental presence of larger and denser MFB clusters on the left of the strand. The 6 mV depolarization of RMP in the CMCs caused a ~30% reduction of I_Na_ availability (product of the gates *h* and *j*), which led to a slower conduction compared to control. Furthermore, isochrones often assumed an oblique orientation reflecting local variations of CV. Also, average dV/dt_max_ was considerably reduced and exhibited large variations with regions of lower dV/dt_max_ corresponding to clusters of endogenous MFBs. Thus, disruption of the uniform CMC architecture by endogenous MFBs results in spatial non-uniformities of RMP and dV/dt_max_ as well as in non-uniform conduction displaying distorted wavefronts.

The dependence of CV, RMP, and dV/dt_max_ on the percentage of endogenous MFBs is illustrated Figures 3C–E. On average, increasing the MFB density from 0 to 40% decreased CV by half, depolarized the RMP from −79 to −69 mV and caused a threefold reduction of dV/dt_max_. These changes were accompanied by enhanced variabilities reflecting increasing levels of heterogeneity caused by the MFBs. Raising the MFB density above 40% resulted in the appearance of spontaneous activity in several computer-generated tissues (1 out of 5 at 45%; 2 out of 5 at 50%); these networks were excluded from the analysis, because the quasi simultaneous excitation of the entire tissue during spontaneous activity caused artificially high CVs. At higher percentages of MFBs, the cellular networks were either spontaneously active or could not be excited anymore.

### Slow conduction in CMC strands with coating MFBs

In previous work, optical mapping of impulse conduction was also conducted in CMC strands coated with cardiac MFBs (Miragoli et al., 2006). In these preparations, we observed that coating MFBs were approximately twice as large as CMCs. Both features were incorporated in our model. Cellular networks measuring 3000 × 80 μm were created with the bottom layer consisting exclusively of CMCs and a coating MFB layer covering the CMC strands to variable extents ranging from 0 to 100% in steps of 10%. The resulting MFB densities thus ranged from 0% (no MFBs) to 50% (full coverage).

**Figure 4:**
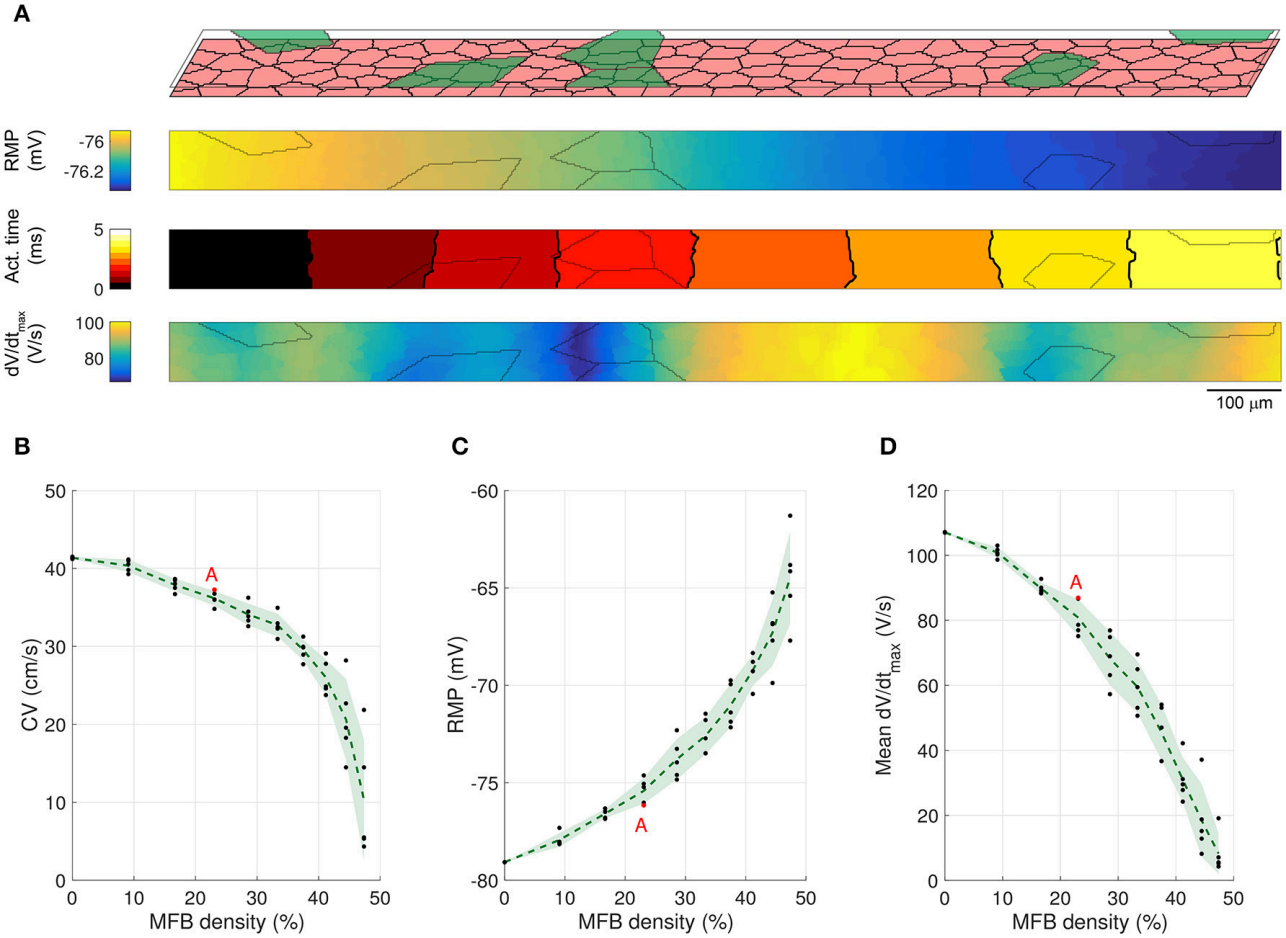
**Conduction in CMC strands with large coating MFBs**. **(A)** Structure of a computer-generated tissue and maps of the corresponding RMP, activation time (isochrone interval: 0.5 ms) and dV/dt_max_. Coating MFBs (MFB density: 23%) are shown in green. In the maps, MFB cell borders are outlined in gray. **(B–D)** Effects of increasing MFB density on CV, mean RMP and mean dV/dt_max_ (black: individual results; green: mean ± SD, *n* = 5; red data point corresponds to the simulated tissue shown in **A**).

A representative simulation of a CMC strand with coating MFBs (23% MFB density) is presented in Figure 4A. Similar to endogenous MFBs, coating MFBs caused a reduction of CV and led to a distortion of the isochrones of activation. This was accompanied by a reduction of RMP with the small gradient present being due to the random assignment of 4 MFBs to the left and 2 MFBs to the right half of the strand. Similarly, local minima of dV/dt_max_ in CMCs correlated with MFB position. As shown in Figures 4B–D, increasing the percentage of coating MFBs reduced CV, RMP, and dV/dt_max_. Furthermore, complete coverage of the CMC strand by MFBs (50% of MFB density) induced spontaneous activity.

**Figure 5:**
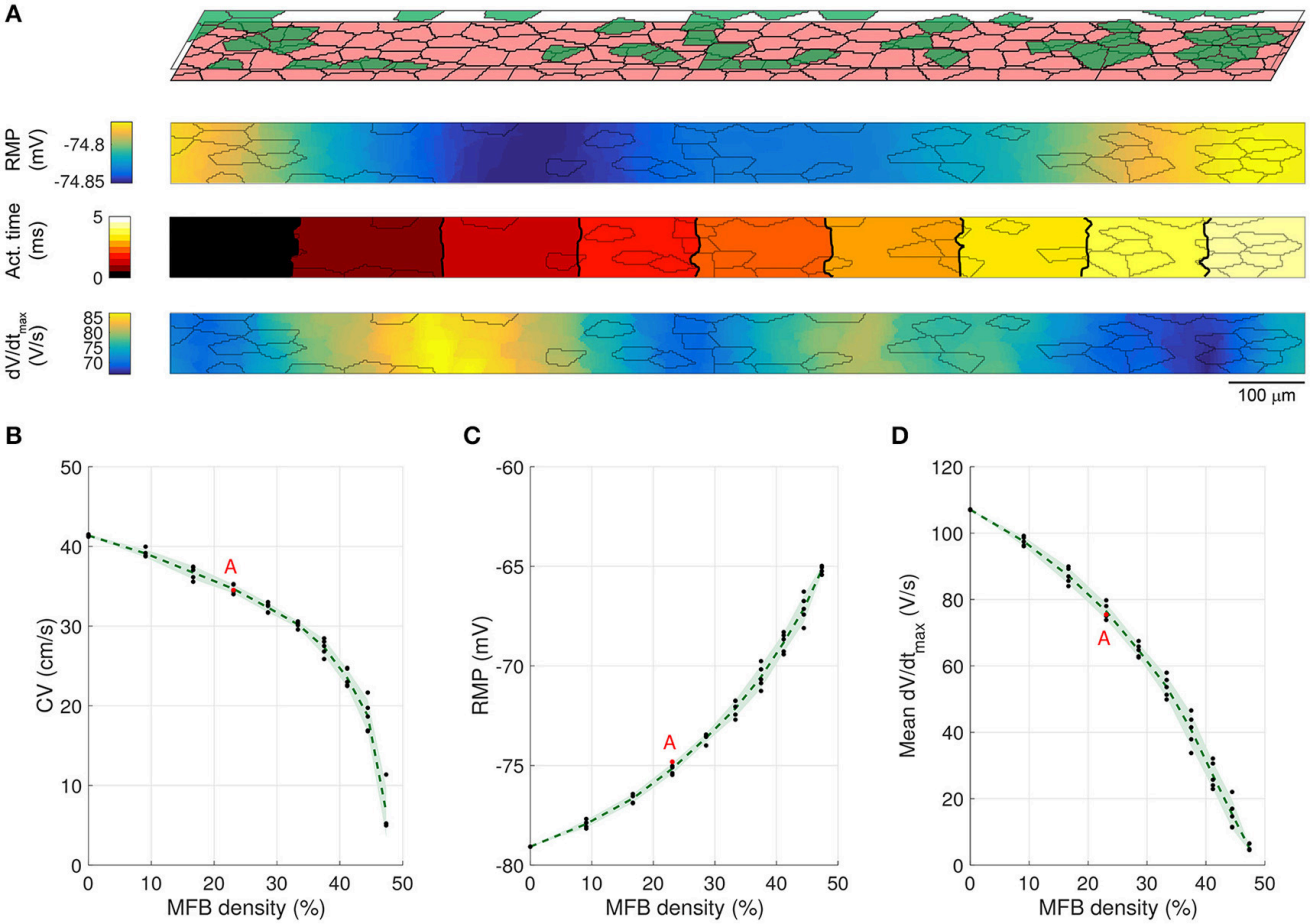
**Conduction in CMC strands with CMC-sized coating MFBs**. **(A)** Structure of a computer-generated tissue and maps of the corresponding RMP, activation time (isochrone interval: 0.5 ms) and dV/dt_max_. Coating MFBs (MFB density: 23%) are shown in green. In the maps, MFB cell borders are outlined in gray. **(B–D)** Effects of increasing MFB density on conduction velocity, mean RMP and mean dV/dt_max_ (black: individual results; green: mean ± SD, *n* = 5; red data point corresponds to the simulated tissue shown in **A**).

To investigate whether the size of the coating MFBs affects conduction characteristics, we performed simulations with MFBs having statistically the same size as endogenous MFBs (i.e., CMC-sized). Figure 5A shows the cellular structure and electrophysiological parameter maps from a representative CMC strand with 30% of its surface covered by CMC-sized MFBs (23% MFB density). Similar to simulations with large MFBs, areas with a larger local density of MFBs exhibited the least polarized RMPs and the slowest upstrokes. At an MFB density of 47%, 4 networks out of 5 were excitable (1 network could not be stimulated) and at an MFB density of 50% (complete MFB coverage), the strands were spontaneously active. Interestingly, the variance of the investigated parameters in five different network realizations was smaller than that observed in models with large coating MFBs (compare Figures 4B–D, 5B–D). This can be explained by the fact that, for a given density, there is a larger number of CMC-sized MFBs seeded over the strand and, therefore, their distribution and effect on the parameters analyzed are more homogenous.

**Figure 6:**
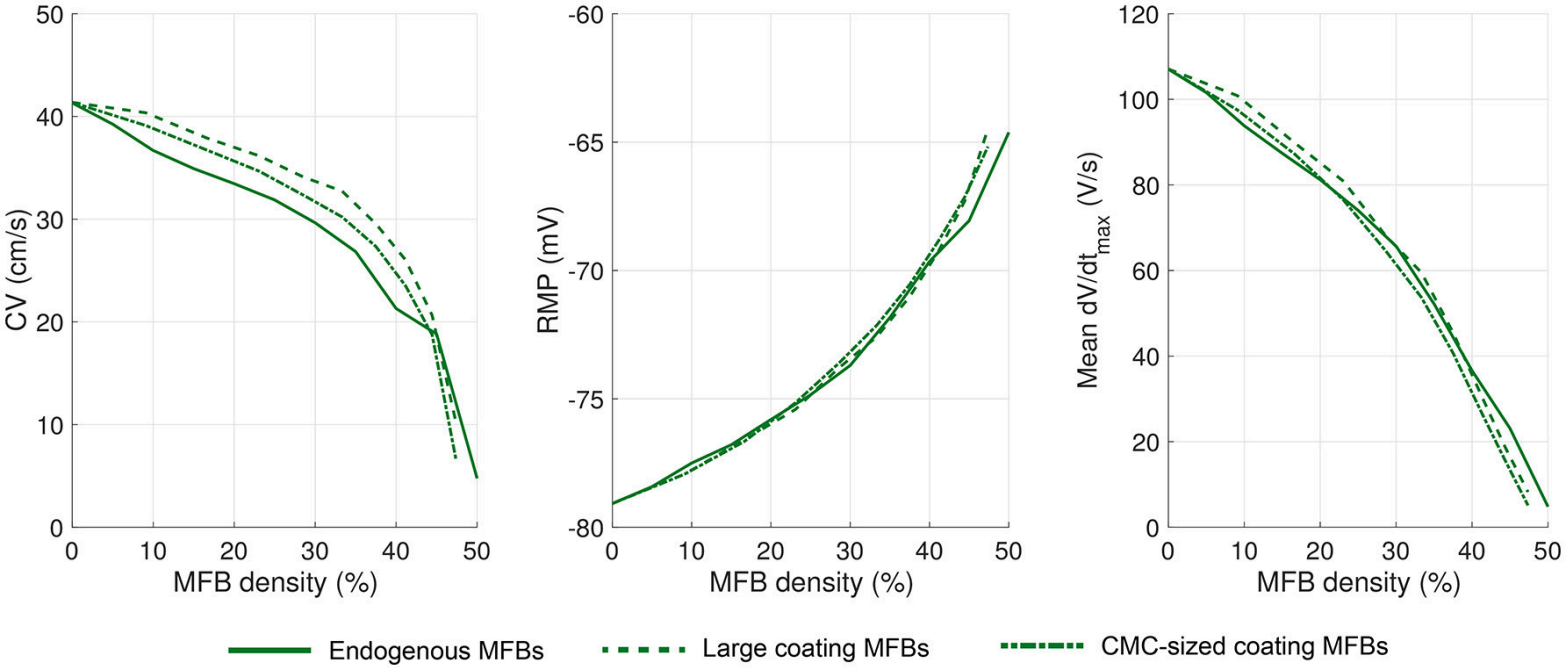
**Comparison of the effects of increasing MFB density on electrophysiological parameters in CMC strands in the three different configurations: endogenous MFBs, large coating MFBs and CMC-sized coating MFBs**. Data are reported as means (*n* = 5 each).

The effects of endogenous MFBs vs. large and CMC-sized coating MFBs are compared in Figure 6. For any given MFB density, endogenous MFBs caused the largest amount of conduction slowing, followed by CMC-sized coating MFBs and large coating MFBs. By contrast, the effects of MFBs on RMP and dV/dt_max_ were comparable among the three different tissue configurations.

### MFBs induce heterogeneous RMP profiles

**Figure 7:**
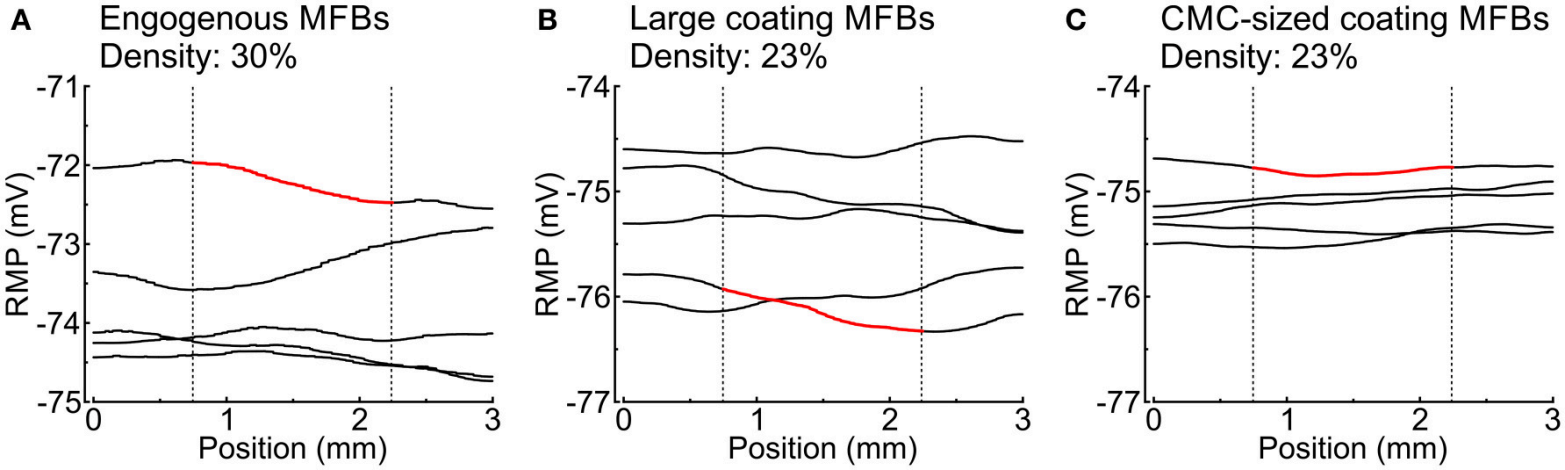
**Spatial profiles of RMP in different tissue realizations along the entire length of simulated strands with (A) endogenous MFBs (density: 30%), (B) large coating MFBs (coverage: 30%; density: 23%) and (C) CMC-sized coating MFBs (coverage: 30%; density: 23%);** *n* = 5 realizations for each case. The profiles corresponding to Figures 3B, 4A, 5A are highlighted in red. Vertical dotted lines denote the central segment used for the analyses in Figures 3–5 (25–75% of the strand).

MFBs induced long-range spatial fluctuations of RMP which appear as gradients in Figures 3B, 4A, 5A. These spatial fluctuations are presented in Figure 7 for all tissue realizations with endogenous as well as large and CMC-sized MFBs (at densities of 30 and 23%, respectively). The spatial RMP profiles appeared inhomogeneous because, at rest, the depolarizing effect of a given MFB did not remain confined to the CMCs in its immediate vicinity but spread to some extent into the syncytium of well-connected CMCs. Since MFBs were distributed stochastically, they tended to form random clusters. Larger clusters and/or regions with a locally larger MFB density produced stronger local depolarizations that reached macroscopic dimensions (mm). Interestingly, large coating MFBs produced stronger spatial RMP variations and larger RMP gradients than CMC-sized MFBs.

### MFB inserts cause long conduction delays

**Figure 8:**
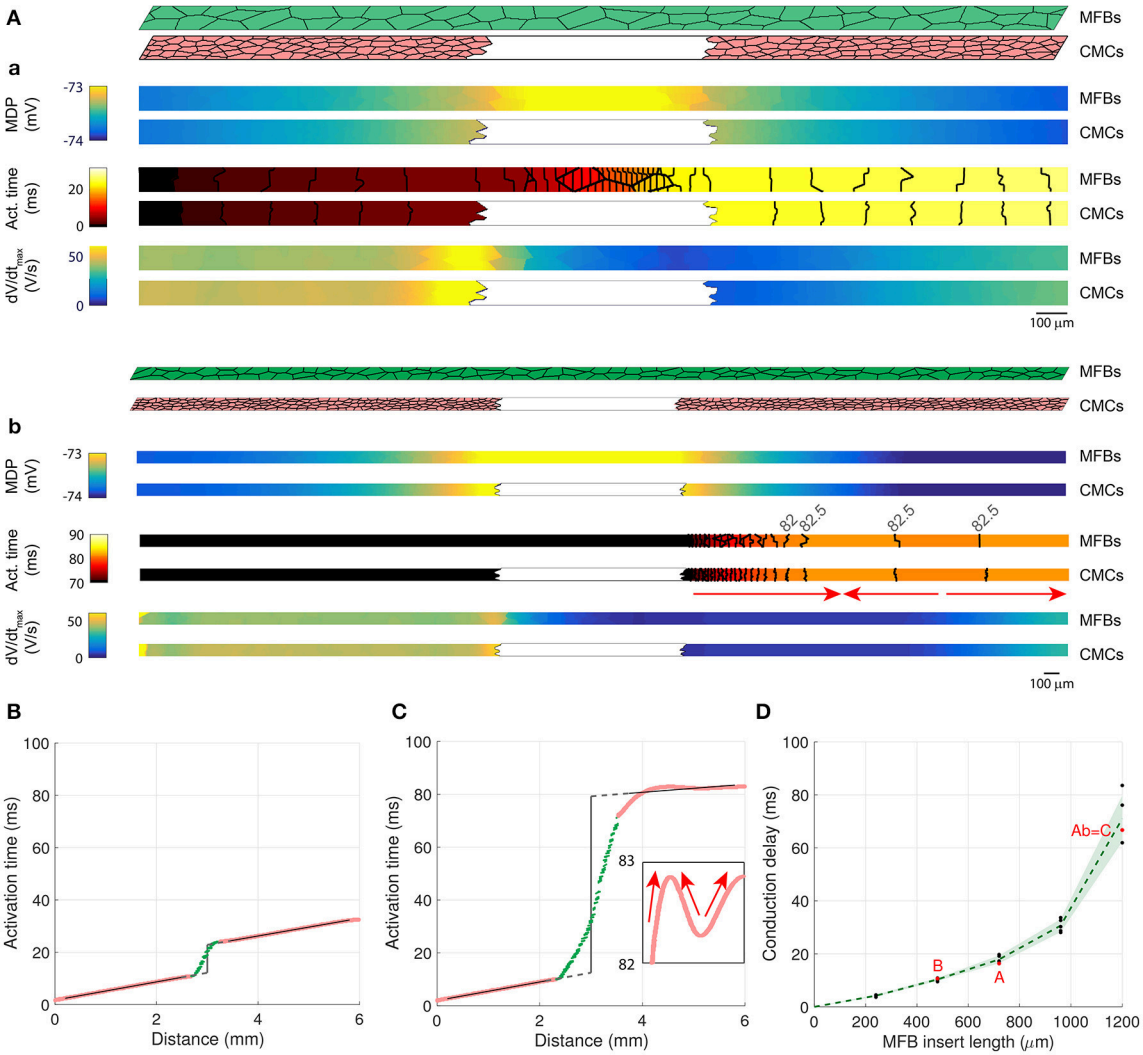
**Two CMC strands linked by a layer of coating MFBs bridging the gap (MFB insert). (Aa,Ab)** Structure of computer-generated tissues and maps of the corresponding minimum diastolic potential (MDP), activation time (isochrone interval: 0.5 ms) and dV/dt_max_ obtained in MFBs (top) and CMCs (bottom). The MFB insert was 720 μm long in **Aa** (12% of total strand length, only the central segment from 25 to 75% is shown) and 1200 μm long in **Ab** (20% of total strand length, the entire strand is shown; the arrows indicate the direction of propagation in the distal segment). **(B)** Activation time along a strand with a 480 μm long MFB insert revealing delayed activation between the two CMC segments (pink) linked by the MFB insert (green). The linear fits in both CMC segments (black) were extrapolated (dashed gray) to the center of the insert to compute the delay (vertical segment). **(C)** Activation time along the strand from **Ab** (MFB insert length; 1200 μm). The inset shows the activation profile in the distal CMC segment on an expanded time scale (the arrows indicate the direction of propagation). **(D)** Dependence of conduction delays on the length of MFB inserts in different realizations of the computer generated tissue (black: individual measurements, green: mean ± SD, *n* = 5; simulations shown in **A–C** are labeled accordingly).

In patterned growth cell cultures in which an MFB bridge (insert) was constructed between two CMC strands, it was observed that MFBs are capable of relaying electrical activation over distances as long as 300 μm (Gaudesius et al., 2003), albeit at the expense of a substantial local propagation delay across the insert (Gaudesius et al., 2003). To investigate conduction in an identical preparation, we generated long CMC strands measuring 6000 × 80 μm interrupted in the center over predefined distances (240–2880 μm). Strands were coated with MFBs (at 100% coverage) that linked the two CMC segments thereby reproducing the essential features of the previous *in vitro* model (Figure 8). The examples presented in Figure 8A (a: insert length: 720 μm; b: insert length: 1200 μm) show that the MFB bridge caused a small depolarization extending electrotonically over several hundreds of μm into the adjacent CMC segments. This depolarization, in conjunction with absence of excitable cells in the gap, resulted in considerable conduction delays across the insert (18 and 67 ms, respectively). Moreover, dV/dt_max_ was extremely low in the insert due to the absence of I_Na_. Conduction delays across inserts were determined from the interval between linear regressions of activation times along the two CMC segments (to avoid border effects, CMC activation data located within 0.2 mm of the insert were ignored; the delay was determined in the center of the insert). Figure 8B illustrates AP propagation over a 480 μm long insert (delay: 11 ms) while Figure 8C illustrates conduction characteristics across the 1200 μm long insert shown in Figure 8Ab (delay: 67 ms). In the case of long inserts (>960 μm), partially retrograde activation in the distal CMC segment was observed (see arrows in Figures 8Ab,C) that reproduced findings obtained in previous *in vitro* experiments. In 3 out of 5 realizations with 1200 μm long inserts, the site of earliest activation was located between the beginning and the end of the distal CMC segment (with anterograde propagation resuming near the extremity, as in Figures 8Ab,C), whereas in the remaining 2 realizations, the site of earliest activation was located at the end at the strand (with retrograde propagation from the end of the strand). These remote activations with retrograde propagation were not caused by spontaneous excitation because no spontaneous activity occurred during the interval 0–200 ms when the stimulus was turned off, and the activation profiles remained similar when the timing of the stimulus was shifted by 5–10 ms.

Notably, dV/dt_max_ showed a local maximum proximal to the insert which is a consequence of the higher resistance of the MFB insert (sealed-end effect). Figure 8D shows the dependence of conduction delays on MFB insert lengths. Delays increased non-linearly with insert length and amounted to 71 ms at 1.2 mm. For longer inserts (1.44 mm and more), conduction failed.

**Figure 9:**
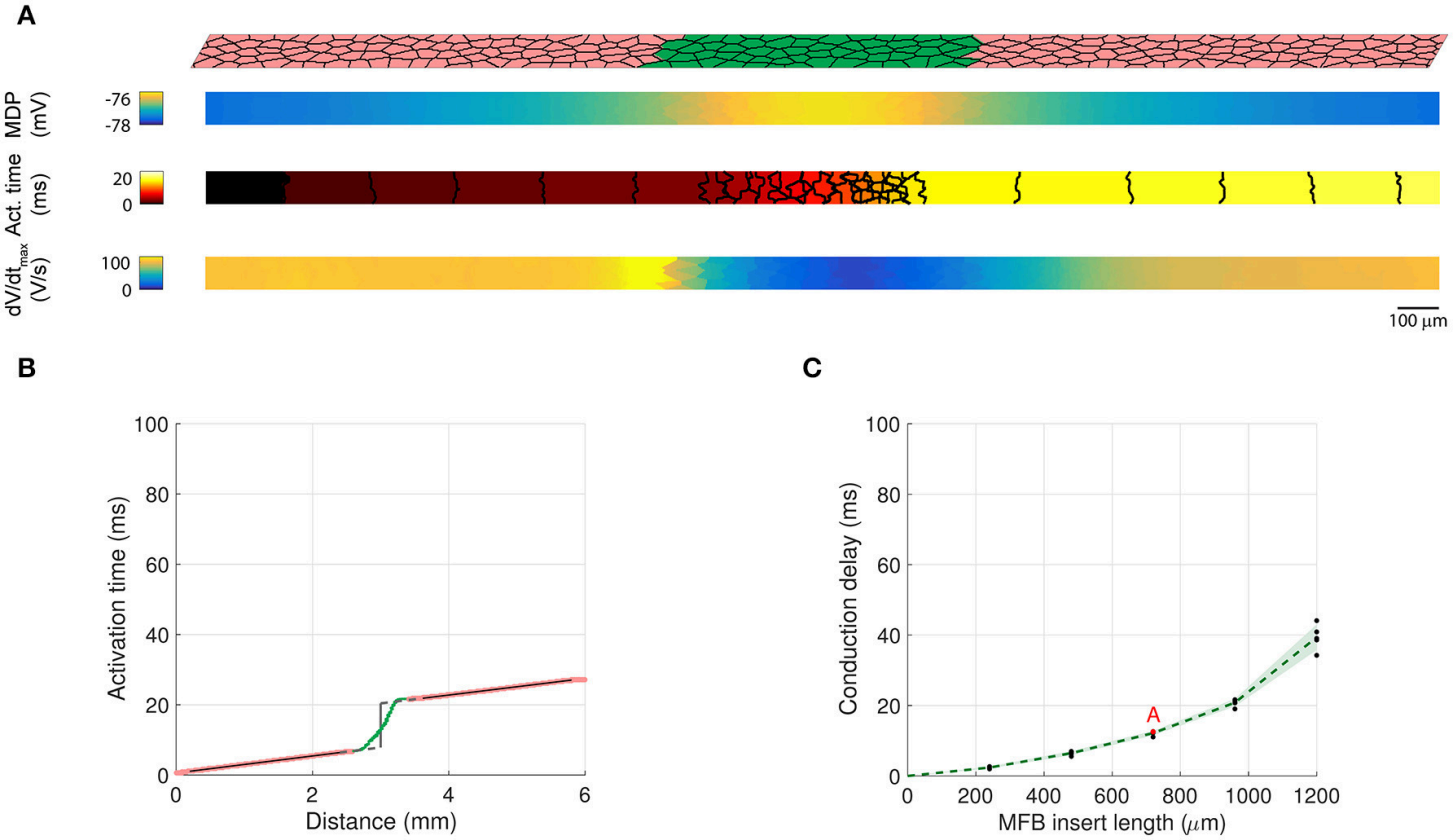
**Two CMC strand segments linked by an insert of CMC-sized MFBs. (A)** Structure of a computer-generated tissue (insert length: 720 μm) and maps of the corresponding minimum diastolic potential (MDP), activation time (isochrone interval: 0.5 ms) and dV/dt_max_. **(B)** Activation time along the structure shown in **A**, showing the activation delay between the two CMC segments (pink) linked by the MFB insert (green). **(C)** Dependence of the conduction delay on MFB insert length in different computer generated tissues (black: individual measurements, green: mean ± SD, *n* = 5; the simulation illustrated in **A** is labeled in red).

In Figure 8, we simulated the exact experimental architecture of the previous *in vitro* study (Gaudesius et al., 2003). However, this architecture combines MFB insertion and MFB coating. Since MFB coating exerts specific effects on conduction (see Figures 4 – 6), we also investigated the situation where MFBs are present exclusively in the inserts (Figure 9). Similar to the simulations with MFB coating (Figure 8), MFB inserts caused conduction delays that increased nonlinearly with insert length. However, for all insert lengths investigated, conduction delays (Figure 9C) were approximately 30% shorter compared to the situation with coating MFBs (Figure 8D). For longer inserts (1.44 mm and more), conduction failed. Thus, the absence of MFB coating of the CMC segments did not cause any major change (20% or more) in the maximum insert length still permitting propagation.

### Untangling the effects of ionic repertoires and intercellular coupling on conduction in CMC strands with endogenous MFBs

Theoretically, the effects of endogenous MFBs on impulse conduction are based on a combination of changes in tissue resistance, tissue capacitance and the lack of excitability of MFBs. To identify the specific contributions of these factors to conduction in our model, we simulated the following two alternative configurations. (i) We applied identical levels of gap junctional coupling to MFB-MFB, CMC-MFB, and CMC-CMC contacts with the goal to isolate the contribution of the different ion channel repertoires of MFBs and CMCs to the observed results (“strongly coupled MFBs”). (ii) We replaced the ion current repertoire of MFBs by that of CMCs (including I_Na_) while maintaining their lower level of coupling with the CMCs, to isolate the specific effects of differences in intercellular coupling on the observed results.

**Figure 10:**
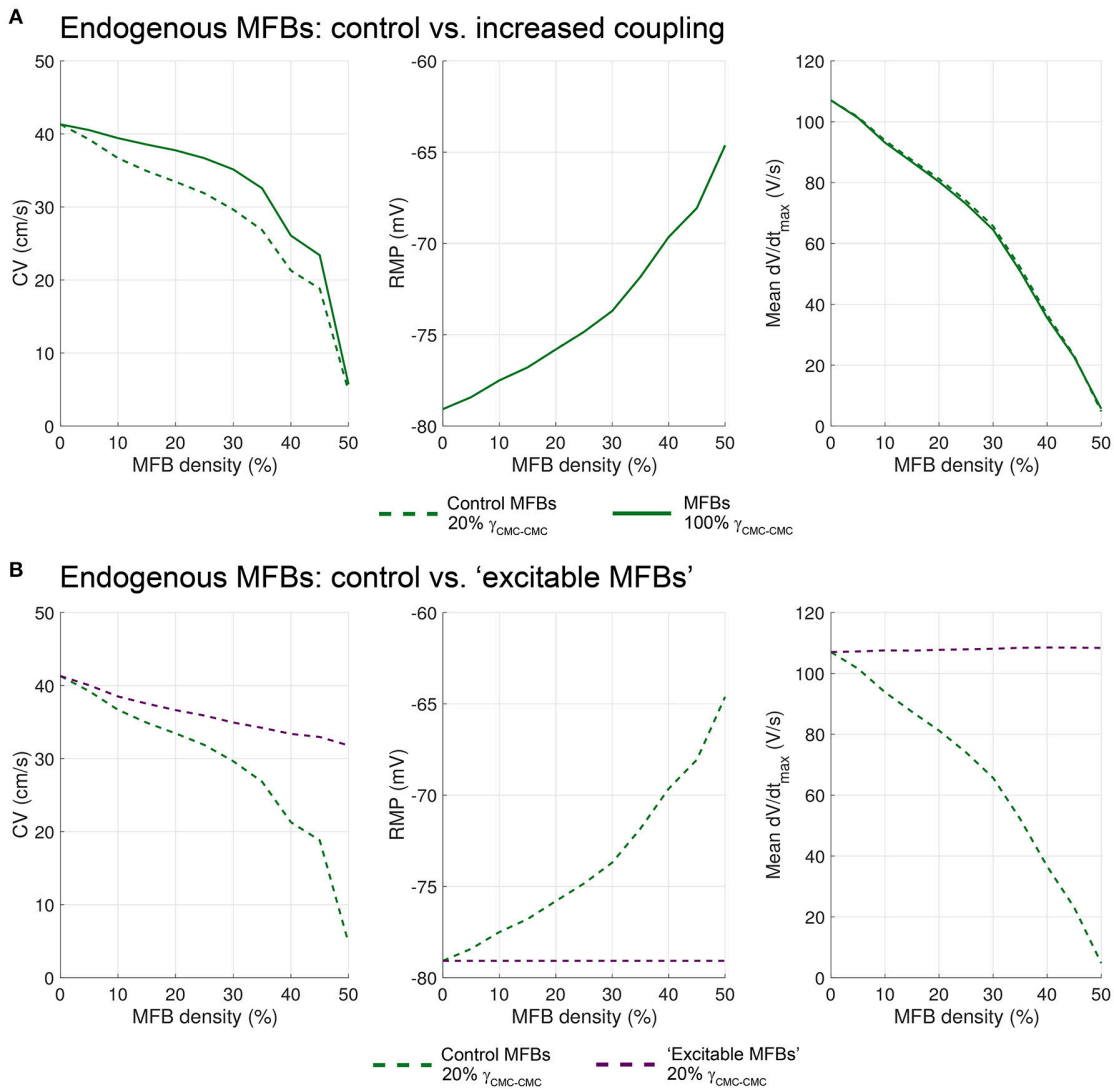
**Differential effects of the intercellular coupling and of the ion channel repertoire of endogenous MFBs on conduction. (A)** Effects of increased coupling (CMC-MFB/MFB-MFB coupling set to normal CMC values, i.e., 100% γ_CMC−CMC_; solid line) as opposed to control levels of coupling (dashed line; data from Figure 3) on CV, RMP and dV/dt_max_. **(B)** Effects of replacing the ion channel repertoire of MFBs with that of CMCs (i.e., generation of “excitable MFBs”; dashed purple lines) as compared to the control situation (dashed green lines; data form Figure 3). Data represent the mean of 5 simulations.

Figure 10A shows that strongly coupled endogenous MFBs substantially increased CV at MFB densities up to 30%. On the other hand, RMP remained largely unaffected resulting in identical availabilities of I_Na_ in CMCs for both coupling conditions and, hence, in comparable dV/dt_max_. The difference in regard to CV is due to the combined effects of intercellular coupling and the capacitive load of coupled MFBs on CV. At MFB densities ≤30%, the slowing effect of normally coupled MFBs was about 2.5 times larger than the slowing effect of strongly coupled MFBs. This observation, together with the finding that the CV curves do not cross, suggests that the increase of the capacitive effect of strongly coupled endogenous MFBs was only modest and was outweighed by the effect of decreased tissue resistance. Thus, the slowing of conduction by normally coupled MFBs was additionally due to the meandering propagation and the axial resistance imposed by the MFBs.

Figure 10B shows that replacing the ion currents of endogenous MFBs by those of CMCs (dashed purple curves) led to an increased CV at all MFB densities compared to control MFBs. In this situation, the RMP of CMCs was not affected by the density of these normally coupled “excitable MFBs.” Consequently, dV/dt_max_ was not decreased because of the presence of a fully available I_Na_ in all cells. In fact, dV/dt_max_ slightly increased, a finding consistent with previous reports on the effects of reduced coupling on dV/dt_max_ in both experiments (Spach et al., 1987) and simulations (Joyner, 1982; Shaw and Rudy, 1997). Thus, “excitable MFBs” did not exert any capacitive loading effect because of the presence of I_Na_. At higher densities of “excitable MFBs,” conduction did not fail and approached 30 cm/s (overall decrease by approximately 25% compared to 41.4 cm/s in control CMC strands). This effect was solely due to increased tissue resistance.

### The depolarizing effect of MFBs prevails when they are situated on top of CMC strands

**Figure 11:**
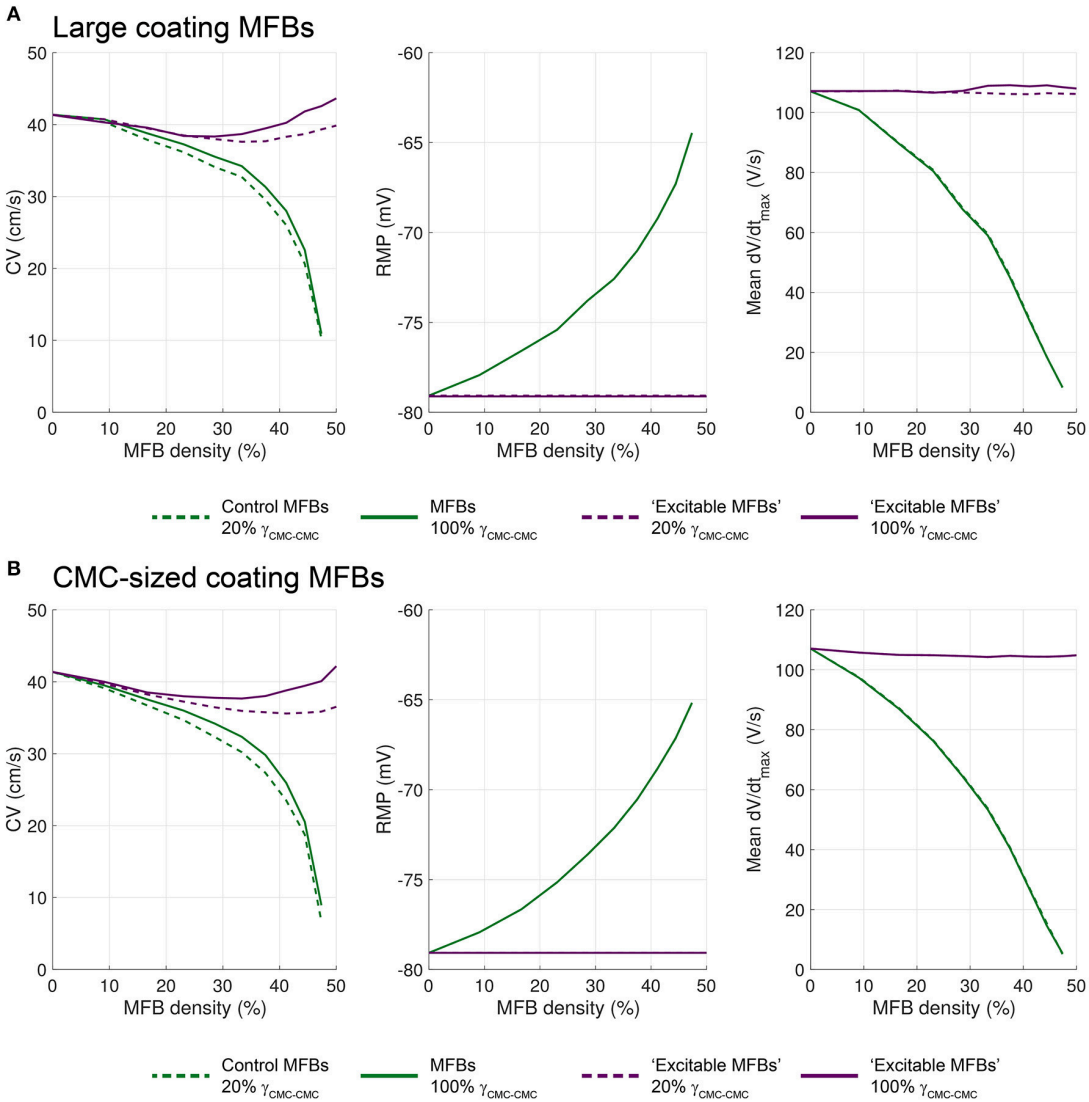
**Effects of gap junctional coupling and of the ionic channel repertoire of coating MFBs on conduction velocity, RMP and dV/dt_**max**_ in CMC strands coated with (A) large MFBs and (B) CMC-sized MFBs**. The dashed green lines correspond to the data from Figures 4, 5. Data corresponding to strong CMC-MFB/MFB-MFB coupling (set to be equal to that between CMCs, i.e., 100% γ_CMC−CMC_) are shown as solid green lines. Data corresponding to replacing the ionic currents of the MFBs with those of CMCs (i.e., “excitable MFBs”) are shown as purple lines (dashed: normal coupling, solid: strong coupling). Data represent the mean of 5 simulations.

The same approach was used to dissect the effects of ionic current repertoires and intercellular coupling in CMC strands with large (Figure 11A) and CMC-sized coating MFBs (Figure 11B). To isolate the effect of the different CMC and MFB ion channel repertoires, MFB coupling was again increased to levels of CMC-CMC coupling (“strongly coupled MFBs”; solid green lines) while the contribution of different intercellular conductances was determined by replacing the ion channel repertoire of MFBs with that of CMC (“excitable MFBs,” dashed purple lines). In addition, we simulated the effects of strongly coupled excitable coating MFBs (solid purple lines). This latter scenario corresponds to a continuous layer of normal CMCs coated with a partially covering second layer of normal CMCs, and examines the specific effect of such an arrangement of CMCs on conduction.

Independent of MFB size, increasing the level of coupling of coating MFBs led to a lesser reduction of CV compared to endogenous MFBs (compare Figure 11 with Figure 10). This difference is likely explained by the fact that, in contrast to endogenous MFBs, coating MFBs are not interfering directly with the path of AP propagation which renders the level of MFB-CMC less important. As with endogenous MFBs, increasing CMC-MFB coupling did not influence RMP or dV/dt_max_, indicating that baseline CMC-MFB coupling levels were sufficient to exert maximal depolarizing effects on adjacent CMCs.

Increasing the density of coating “excitable MFBs” (dashed and continuous purple lines, respectively) exerted only small changes on CV and had no appreciable effect on RMP and dV/dt_max_. This again indicates that the depolarizing effect of MFB prevails when they are seeded on top of CMC strands. Interestingly, these changes were biphasic: increasing the coverage by “excitable MFBs” first caused a decrease of CV, followed by an acceleration of conduction.

**Figure 12:**
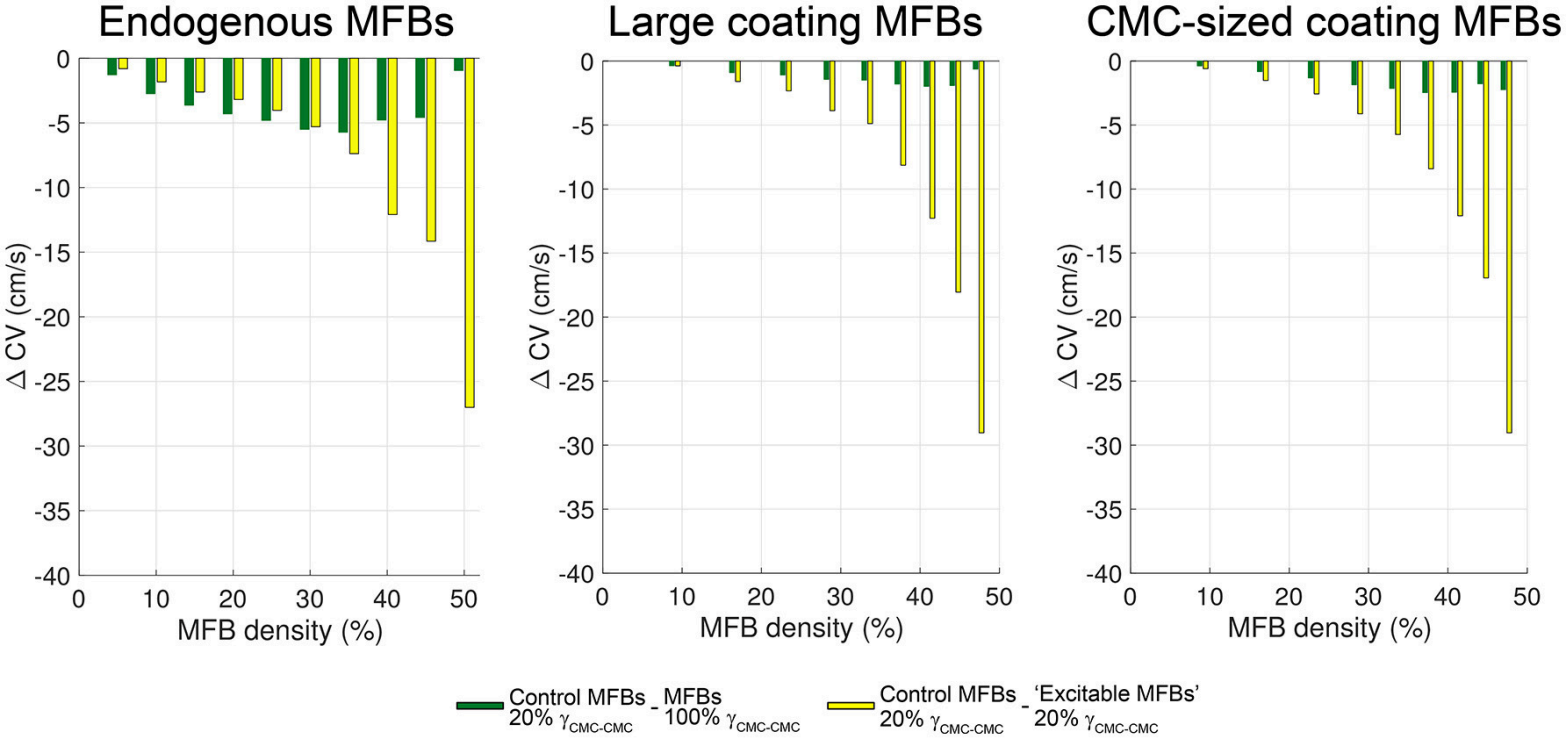
**Relative contributions of coupling strength and electric phenotype of MFBs to impulse conduction slowing**. For all three types of CMC-MFB arrangement, the differences (ΔCV) between control CVs and CVs with strongly coupled MFBs are displayed in green, and the differences between control CVs and CVs with “excitable MFBs” are shown in yellow.

A summary of the relative contributions of gap junctional coupling and electrophysiological characteristics of MFBs to slow conduction in heterocellular tissue preparations is shown in Figure 12. Data are based on the findings presented in Figure 10 and Figure 11 with green bars indicating the contribution of gap junctional coupling (moderate compared to CMCs) and yellow bars indicating the contribution of the specific electrophysiological phenotype of MFBs (non-excitable cells with a reduced membrane potential) to slowing of conduction. In CMC strands with endogenous MFBs, the resistive effect of MFBs dominated the reduction of CV in the range 5–30% MFBs while, at higher densities, conduction slowing was primarily due to effects related to the electrical phenotype of MFBs (green vs. yellow bars in the left panel of Figure 12). By contrast, in strands with either large or small coating MFBs, conduction slowing induced by MFBs was dominated by effects related to the electrical phenotype of MFBs over the full range of MFB density (Figure 12, middle and right panels). Thus, the arrangement of the MFBs (endogenous vs. coating) influenced the relative importance of the mechanisms responsible for MFB-dependent conduction slowing.

### The unexcitable nature of MFBs is the cause of conduction delays across MFB inserts

**Figure 13:**
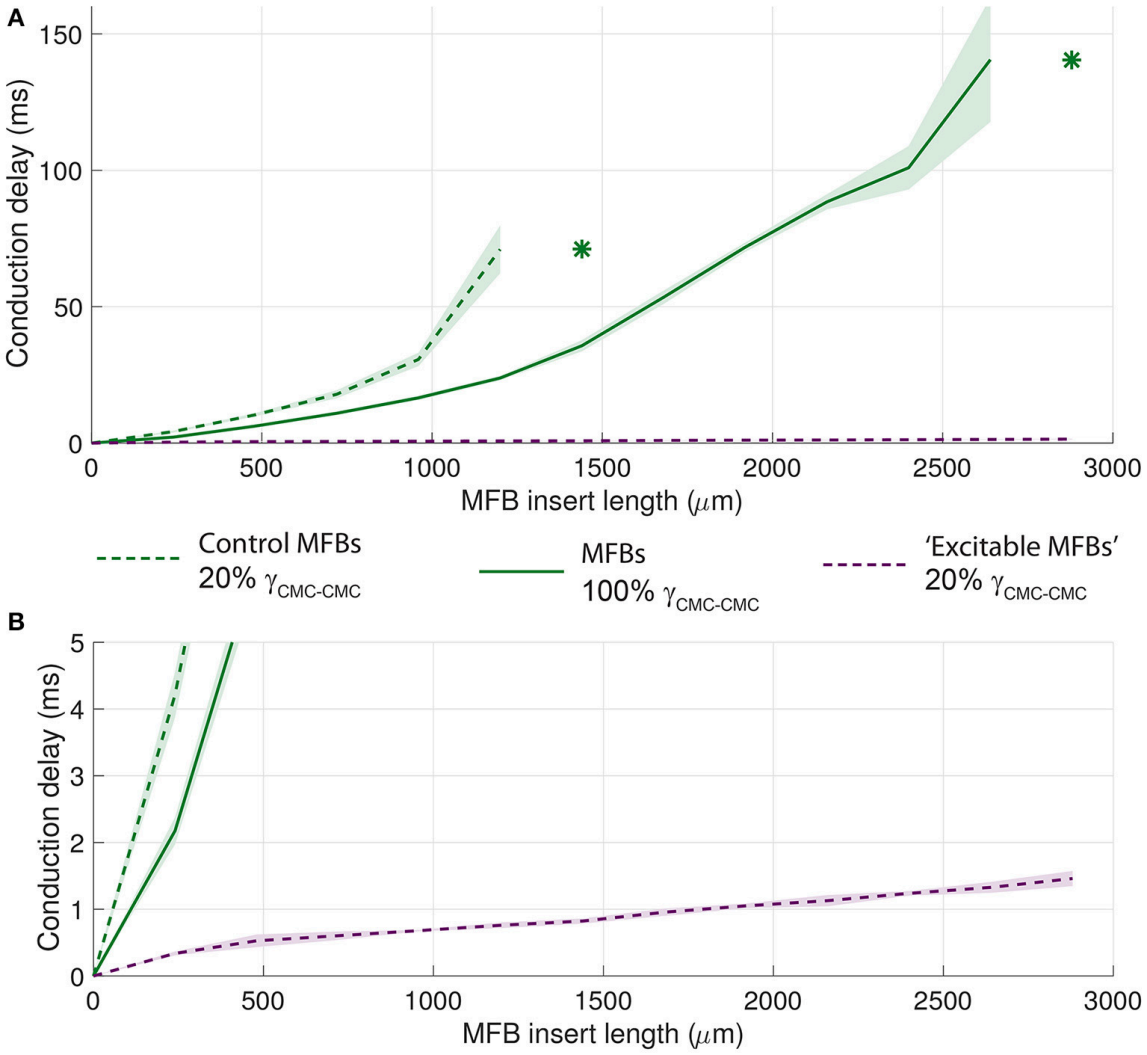
**Effects of the level of MFB-CMC coupling and of the ionic channel repertoire of MFBs on conduction delays across MFB inserts. (A)** The dashed green lines correspond to the control data from Figure 8. Data corresponding to a strong level of CMC-MFB/MFB-MFB coupling (set to be equal to that between CMCs) are shown as solid green lines. Data corresponding to replacing the ionic currents of the MFBs with those of CMCs (i.e., “excitable MFBs”) are shown as dashed purple lines. Data are reported as mean ± SD (*n* = 5). Asterisks denote the first insert length at which intermittent block occurred. **(B)** Same as **A**, but with a rescaled ordinate to emphasize the evolution of conduction delays with longer inserts of normally coupled “excitable MFBs” (purple in both **A,B**).

Finally, analogous strategies were used to identify the primary determinants of conduction delays across MFB inserts (Figure 13). When the MFB ionic repertoire was replaced with that of CMCs (“excitable MFBs,” dashed purple line), the conduction delay decreased by almost two orders of magnitude and conduction block was absent which suggests that the effect of intercellular coupling is minor compared to the effect of the absence of excitability in the insert. However, introducing strongly coupled MFBs (solid green line) reduced the conduction delay for all insert lengths compared to control conditions (dashed green line). Moreover, conduction block occurred only with longer MFBs inserts (>2640 μm). This result indicates that, in the presence of unexcitable cell inserts, intercellular coupling modulates conduction delays and the propensity to conduction block.

## Discussion

In this study, we developed a detailed cell-based model of fibrotic cardiac tissue that combines CMCs and MFBs, i.e., two cell types expressing different levels of connexins and exhibiting intrinsically different repertoires of ionic currents. Three cellular arrangements were investigated: CMC strands with endogenous MFBs, CMC strands coated with MFBs, and CMC strands with MFB inserts. The model reproduced previous *in-vitro* findings in regard to MFB-induced slow conduction, reduction of upstroke velocities and depolarization of CMCs with high fidelity. Mechanistically, the model revealed that the specific electrophysiological phenotype of MFBs lacking I_Na_ and exhibiting a depolarized membrane potential is one of the main drivers for conduction slowing. Interestingly, the simulations showed that randomly positioned MFBs generate long-range spatial variations of RMP. Moreover, the model showed that, for any given MFB density, endogenous MFBs exert a stronger effect on CV than coating MFBs because they act both as a resistive obstacle to conduction and as a source of depolarizing current flow to CMCs.

The effects of MFBs on the propagated AP depend on multiple factors. Besides ionic currents of the two cell types and MFB density, they depend on the coupling conductance between CMCs and (myo)fibroblasts (G_CMC−MFB_) and the capacitance of MFBs (C_MFB_). Jacquemet and Henriquez (2008) described the following three coupling regimes for G_CMC−MFB_ and the ratio G_CMC−MFB_/C_MFB_. In the low coupling regime (defined as <0.25 nS for a 6.3 pF fibroblast, i.e., G_CMC−MFB_/C_MFB_ < 0.04 nS/pF), the G_CMC−MFB_ is sufficient to depolarize the RMP of CMCs and to cause supernormal conduction with increasing MFB density, but the membrane potential of MFBs follow slowly (or hardly at all) that of CMCs, especially during the AP upstroke. During conduction, the MFB thus behaves as a resistive sink determined by G_CMC−MFB_ (Jacquemet and Henriquez, 2009). In the intermediate coupling regime (0.25–8 nS for a 6.3 pF fibroblast, i.e., G_CMC−MFB_/C_MFB_ of 0.04–1.3 nS/pF), the depolarizing effect on myocyte RMP saturates and both G_CMC−MFB_ and C_MFB_ influence CV and the shape of the action potential. In the strong coupling regime (>8 nS for a 6.3 pF fibroblast, i.e., G_CMC−MFB_/C_MFB_ > 1.3 nS/pF), the depolarizing effect on myocyte RMP is saturated, the membrane potential of the MFBs follows closely that of the CMCs and the effects on CV are dominated by C_MFB_. MFBs thus behave as capacitive loads. The effects of MFBs on CV and the action potential waveform then hardly change with a further increase of coupling, and, in this regime, supernormal conduction is not observed when MFB density is increased.

It is thus important to consider what coupling regime pertains to our engineered preparations and our model. Considering an endogenous MFB capacitance in the range of 20 pF and a single connexin 43 (Cx43) channel conductance of 96 pS (Valiunas et al., 1997), the low and strong coupling regimes would correspond to <8 and >270 channels, respectively. Our previous immunohistochemical studies (Gaudesius et al., 2003; Miragoli et al., 2006) showed a clear pattern of both Cx43 and Cx45 labeling between CMCs and MFBs, compatible with hundreds of channels and thus suggesting a strong coupling regime. Moreover, measurements of gap junctional resistance in CMC-MFB pairs (Salvarani et al., 2012) are in the order of 74 nS (for a contact length of 50 μm), which is again compatible with a strong coupling regime >3 nS/pF. Whether the regime is also strong in the intact heart is still elusive. In the simulations, the strong coupling regime was reflected by the fact that dV/dt_max_ in MFBs was >50% of that in CMCs (Figure 3B). Thus, the capacitive loading effect of MFBs is the second main driver for conduction slowing.

### Comparison with previous work

The detailed tissue model replicates the principal conduction characteristics observed in experiments (Miragoli et al., 2006) and provides insights at a cellular level into discontinuous propagation characteristics in heterocellular tissues. Endogenous MFBs induced arrhythmogenic slow conduction and ectopic activity when present at sufficiently high densities, which illustrates that the electrophysiology of cardiac tissue can be substantially modulated by electrotonically coupled MFBs. Consistent with experimental findings, we found that CV was about half of that in pure CMC strands at 40% MFB density (Miragoli et al., 2006). In the presence of coating MFBs, CV, and upstroke velocity depended on MFB density (Miragoli et al., 2007). General effects of MFBs on cardiac electrophysiological properties observed in the present study are comparable with effects reported previously by other investigators (MacCannell et al., 2007; Jacquemet and Henriquez, 2008; Sachse et al., 2008; Xie et al., 2009a; Nayak et al., 2013).

While slow conduction is important for the induction and the perpetuation of reentry, further effects of MFBs may contribute to arrhythmogenesis, which we did not address in our simulations. CMC-MFB coupling was suggested to potentiate reentry by inducing post-repolarization refractoriness (Xie et al., 2009a) and discordant action potential duration (APD) alternans (Xie et al., 2009b). Moreover, it was shown that triggered activity due to early afterdepolarizations is potentiated by CMC-MFB coupling during oxidative stress or hypokalemia (Morita et al., 2009; Nguyen et al., 2012).

By causing proarrhythmic conduction slowing via depolarization of CMCs, MFBs are usually considered to be detrimental for heart function. Interestingly, however, we found no conduction blocks in the present simulations (unless MFB density was increased above 40%) and in the corresponding previous experiments (Miragoli et al., 2006). This absence of conduction block contrasts with the results of a previous experimental-computational study on conduction in mixtures of wild-type and connexin 43 knock-out (Cx43KO) CMCs, in which it was observed that conduction frequently blocked for Cx43KO CMC densities in the range 20–80% (Prudat and Kucera, 2014). The scenario of mixed wild-type and Cx43KO corresponds to endogenous MFBs being deprived of gap junctions. Such completely uncoupled MFBs would induce gaps in the tissue structure resulting in sites of current-to-load mismatch that exhibit a high propensity for block. Based on this consideration we postulate that a certain level of CMC-MFB coupling may in fact be desirable as it prevents highly arrhythmogenic conduction blocks, at the expense of slightly reducing CV.

### Endogenous vs. coating MFBs

Because our model accounts for the specific cellular tissue architecture, we were able to directly compare the effects of endogenous vs. coating MFBs. For the case of endogenous MFBs, two mechanisms contributed to slow conduction: the progressive depolarization of CMCs, which caused partial inactivation of I_Na_, and the low level of coupling between CMCs and the endogenous MFBs, which increased the tissue series resistance, thereby limiting axial flow of depolarizing current. Hindrance of axial current flow was absent in CMC strands coated with MFBs. Indeed, in case of coating MFBs, slow conduction results from the depolarizing effect of MFBs and not from changes in gap junctional coupling within the underlying CMC layer, since MFBs are not directly located in the path of propagation but are positioned in parallel. These results are in agreement with the previous observations of Xie et al. (2009a), although these authors used a tissue model with a coarse discretization of 25 μm. Simulations that take distinct cellular arrangements into account are thus essential to reveal the subtle mechanistic differences that arise on the basis of the cellular architecture of tissues composed of CMCs and MFBs as is likely to be the case in the fibrotically remodeled heart. There, myofibroblasts connected sideways to single myocytes would correspond to the coating MFB model (parallel) whereas MFBs forming small connected bridges between myocytes would correspond to the endogenous MFB model (in series).

As shown in Figure 7, stochastically seeded MFBs resulted in a spatially heterogeneous RMP that gave rise to voltage gradients extending to the millimeter range (Figures 3B, 4A, 5A). Noteworthy, the RMP variations were stronger for large MFBs than for CMC-sized MFBs. This can be explained by the tendency of large MFBs to form equally large clusters that are more distant from each other because there are less MFBs per unit length for any given MFB density. With large MFBs, larger RMP variations also explain the larger variability of CV and dV/dt_max_ (compare Figures 4, 5). When I_Na_ of CMCs is partially inactivated by depolarization, RMP gradients may be associated with gradients of excitability which potentiate conduction blocks, e.g., during pharmacological interventions or rapid pacing. Thus, large and non-uniformly distributed MFBs may contribute to arrhythmogenesis more than small MFBs.

An interesting difference can be observed when the ionic repertoire of MFBs is switched to that of CMCs (= “excitable MFB”) in the endogenous vs. coating MFB arrangements (Figures 10B, 11, purple lines). For endogenous “excitable MFBs,” CV decreased monotonically with MFB density (Figure 10B). By contrast, increasing the density of coating “excitable MFB” resulted in a biphasic CV response (Figure 11). The initial slowing can be explained by the fact that a few coating “excitable MFBs” represent recurrent current loads, akin to branching structures known to slow conduction (Kucera et al., 1998; Kucera and Rudy, 2001). In contrast, when the coverage by “excitable MFBs” is high, the AP propagates through two parallel interconnected layers. Since the randomly generated cellular arrangement is different in the two layers, the electrotonic current can find additional alternate pathways through the other layer to circumvent sites of localized resistance at cell-cell junctions. This explanation is in line with the result of previous studies in which it was shown that transverse/lateral uncoupling slows conduction (Hubbard et al., 2007; Prudat and Kucera, 2014). In our model, the reverse occurred as a layer of coupled coating “excitable MFBs” accelerated conduction.

### How do MFBs induce supernormal conduction?

In our previous *in vitro* study (Miragoli et al., 2006) of CMC strands with endogenous MFBs, it was observed that the behavior of CV was biphasic with increasing MFB densities: CV first slightly increased when MFB density was increased from 0 to 10% and then steadily decreased with a further increase of MFB density. This initial increase was attributed to supernormal conduction, whereby the initial slight depolarization by a low number of MFBs brings the RMP of CMCs closer to the threshold of I_Na_ activation (thus accelerating conduction), whereas further RMP depolarization causes partial I_Na_ inactivation (thus slowing conduction). While our CMC model (based on Luo-Rudy I_Na_ dynamics) can reproduce supernormal conduction caused, e.g., by a change of [*K*^+^]_*o*_ (Luo and Rudy, 1991; de Lange and Kucera, 2009, 2010), it failed to reproduce supernormal conduction caused in experiments by a small density of MFBs (Miragoli et al., 2006). This observation is not surprising, since our model is characterized by a high coupling regime (see above). Thus, the passive capacitive loading effect of MFBs (lacking I_Na_) and, in the case of endogenous MFBs, the increased axial resistance, exerted effects on CV that offset the small supernormal acceleration of conduction caused by a slight RMP depolarization. Furthermore, the RMP of CMCs at which peak supernormal conduction occurs when using the LR1 I_Na_ kinetics is near −77 mV (Shaw and Rudy, 1997; Xie et al., 2009a), which is very close to our model RMP of −79 mV. The supernormal effect due to depolarization is therefore expected to be minimal and easily masked by other factors, such as the capacitive loading effect of the MFBs.

Additionally, the discrepancy between model and experiment may be explained by humoral interactions between MFBs and CMCs that affect ion channel/connexin expression in a density dependent manner such as to provide the basis for a biphasic change in CV. Alternatively, coupling of individual MFBs to CMCs may increase over-proportionally at higher MFB densities as migration is increasingly hindered thereby providing more possibilities to establish stable gap junctions. Clearly, further experiments are needed to answer this specific question.

### MFB inserts

Consistent with previous experiments (Gaudesius et al., 2003), MFB inserts of increasing length caused highly discontinuous conduction with increasing conduction delays. When the ionic repertoire of MFBs was replaced by that of CMCs, these delays strongly decreased, revealing that the unexcitable nature of MFBs was the main cause of these delays. When conduction was simulated across MFB inserts without coating the proximal and distal CMC strands with MFBs (a hypothetical situation that does not correspond to the *in vitro* experiments), the conduction delays were shorter but the longest insert length still permitting propagation was not substantially different. Interestingly, and in agreement with experiments (Gaudesius et al., 2003), partially retrograde activation in the distal CMC segment was observed with long MFB inserts and large conduction delays (see Figure 8C). This retrograde activation can be explained by the fact that, with long MFB inserts, I_Na_ is substantially inactivated during the slow sub-threshold depolarization occurring in the distal CMC segment in the immediate vicinity of the insert (note the low dV/dt_max_ in the distal CMC segment in Figures 8A, 9A), while it is less inactivated at more remote locations in that segment. Thus, when the conduction delay is extremely long, the earliest distal activation occurs at a site located beyond the beginning of the distal CMC strand or even at its end, and retrograde propagation is essentially electrotonic. These mechanisms are in line with those reported by Cabo and Barr (1992), who modeled conduction across a site of increased gap junctional resistance separating two myocardial fibers of varying length. When the distal segment was short (4 mm), the earliest distal activation occurred at its end, and when the distal segment was long (6 mm), this site was located inside that segment. Interestingly, the maximal conduction delay was shorter in presence of a long distal segment. These observations indicate that propagation across long MFB inserts and the site of earliest distal activation strongly depend on the kinetics and voltage-dependence of I_Na_ inactivation as well as on the length of the distal CMC segment.

While model and experiment produced virtually identical results in regard to maximal conduction delays before block (~70 ms), conduction delays in the model were smaller than those observed experimentally for any given insert length. Moreover, maximal insert lengths still supporting conduction were longer in the model than in experiments. This discrepancy is likely explained by the fact that, in the simulations, gaps were completely filled with MFBs while, in experiments, MFBs typically failed to fill the entire width of the gap, thereby causing an increase in axial resistance. Additionally, it cannot be ruled out that the conductance between MFBs and between MFBs and CMCs may have been lower than what we assumed in our simulations. This possibility needs to be addressed by future experiments in homo- and hetero-cellular cell pairs.

### Implications

The simulations that we conducted pinpoint different mechanisms that could be targeted to counteract the arrhythmogenic consequences of age- and disease-related cardiac fibrosis. First, strategies could be developed to enhance the coupling between cardiomyocytes and fibroblasts/myofibroblasts. Such approaches could be based on drugs acting directly on gap junctions (e.g., rotigaptide Nielsen et al., 2012) or indirectly by suitable cytokines or humoral messengers. In this context, transforming growth factor β_1_, which we showed before to increase CMC-MFB coupling (Salvarani et al., 2012) or agents acting specifically on the downstream signaling pathway may hold promise. Second, our simulations indicate that approaches aiming at rendering fibroblasts/myofibroblasts excitable would be worth considering. Contrary to our electrophysiological observations, some researchers have reported presence of I_Na_ in human cultured fibroblasts (Chatelier et al., 2012; Poulet et al., 2016). It would thus be of clinical interest to investigate in greater detail what mechanisms lead to the expression of cardiac Na^+^ channels in fibroblasts/myofibroblasts, with the prospect of a clinical application. Third, it should be of interest to investigate whether a normal membrane polarization could be reinstated in these cells, e.g., by targeted genetic therapies with the inward rectifier K^+^ current. Finally, it is worth adding that the findings of this study can be transferred to other situations where CMCs are coupled to non-CMCs having an altered ion channel repertoire such as that exhibited, e.g., by stem cells.

### Limitations

Questions may arise why we modified the LR1 model instead of using a previously published neonatal cell model (e.g., Wang and Sobie, 2008; Korhonen et al., 2009), and why our validation was based on total steady state I–V curves of CMCs and not on individual currents. The model by Wang and Sobie is a model of the neonatal mouse ventricular action potential and thus pertains to another species. The model by Korhonen et al. was developed for investigating Ca^2+^ signaling and diffusion in spherical cells, whereas we focused on propagation in non-spherical cells. Nevertheless, we adopted the I_*K*1_ formulation of Korhonen et al. because it reproduces the experimental I–V curves obtained in CMCs better than the I_*K*1_ formulation of the LR1 model. Using total steady state I–V curves was important for comparing *in silico* findings to experimental data because, in particular near the RMP, models frequently yield I–V curves that deviate substantially from experimental observations. Future determinations of ion currents contributing to total I–V curves in CMCs will be required to reach a comprehensive understanding of electrotonic interactions between MFBs and CMCs.

Similar to CMCs, the MFB formulation used did not incorporate individual ionic currents but was based on the experimentally determined mean global current-voltage relationship. More refined models of fibroblasts exist (MacCannell et al., 2007; Jacquemet and Henriquez, 2008; Sachse et al., 2008), but these models did not reproduce well the current-voltage relationships observed in our myofibroblasts. For this reason, we used our own implementation of MFB currents based on steady state I–V relationships. While time-dependent MFB currents may affect repolarization, their gating involves timescales that are much longer that the AP upstroke and thus the gating of such currents is unlikely to substantially affect propagation. Although, our MFB formulation lacked this dynamical aspect, it reproduced the influence of MFBs on RMP and the resulting perturbation of impulse propagation quite accurately.

We noted that cell-cell contacts assumed staircase-like shapes after discretizing the tissue. While such shapes are reminiscent of intercalated discs, these shapes do not reflect MFB-CMC contacts *in vitro*. An alternative approach for preventing the appearance of staircase-like shapes consists of using a finite element method as described by Kim et al. (2010). However, since individual cells are almost isopotential during depolarization, such fine detail would probably have only a marginal influence on the overall behavior of propagation. This assumption is supported by a study with a microstructure model similar to ours (Gouvêa de Barros et al., 2015), in which it was shown that representing every cell by a single node has only minor effects on the spread of excitation.

Finally, our simulations did not take into account intercellular variabilities of ion current densities, an aspect which may bear a significant importance in novel modeling paradigms (Mirams et al., 2016). These diverse considerations open prospects for further studies.

## Author contributions

FJ developed simulation code, performed the simulations, analyzed the data and wrote the manuscript. AM designed the confocal imaging experiments, acquired and analyzed the images, and wrote the manuscript. SR designed the study and wrote the manuscript. JK designed the study, developed simulation code and wrote the manuscript.

## Funding

This work was supported by the European Network for Translational Research in Atrial Fibrillation (EUTRAF, 261057 to SR) of the FP7 Program of the European Union, the Leducq Foundation (to SR), and the Swiss National Science Foundation (31003A-156738/1 to JK, 138297 to SR).

### Conflict of interest statement

The authors declare that the research was conducted in the absence of any commercial or financial relationships that could be construed as a potential conflict of interest.
